# Structural basis of regioselective double halogenation of the β-carboline tryptoline by the single-component halogenase AetF

**DOI:** 10.1107/S2059798326005954

**Published:** 2026-06-22

**Authors:** Simon Bork, Hendrik J. Horstmeier, Bjarne Scharkowski, Hartmut H. Niemann

**Affiliations:** a Structural Biochemistry, Department of Chemistry Bielefeld University Universitätsstrasse 25 33615Bielefeld Germany; b Organic and Bioorganic Chemistry, Department of Chemistry Bielefeld University Universitätsstrasse 25 33615Bielefeld Germany; McGill University, Canada

**Keywords:** tryptophan halogenases, bromination, iodination, regioselectivity, site selectivity, flavoenzymes, non-natural substrates, biocatalysis

## Abstract

Flavin-dependent halogenases (FDHs) offer a biocatalytic route to regioselective halogenation of aromatic substrates under mild conditions. AetF, a rare self-sufficient single-component FDH, catalyzes sequential dibromination of tryptophan and has a broad substrate scope. Here, we report the crystal structure of AetF in complex with the non-native substrate tryptoline, a tricyclic β-carboline with pharmacological relevance. The structure reveals that tryptoline binds in a pose analogous to the natural AetF substrate l-tryptophan, positioning its C6 atom for initial halogenation. Enzymatic assays confirmed AetF-mediated monobromination and dibromination of tryptoline, yielding 6,8-dibromotryptoline as the final product. In accordance with the observed binding pose of the substrate tryptoline, the monobrominated initial product was almost exclusively 6-bromotryptoline. A crystal structure of AetF in complex with the minor monobrominated product 8-bromotryptoline provides insight into the effect of bromine substituents in different positions on the binding affinity and into potential pathways to the formation of 6,8-dibromotryptoline. These findings define the structural basis of site-selective halogenation of a non-native substrate by AetF and provide structural guidance for enzyme engineering.

## Abstract 2

A structure of the flavin-dependent single-component tryptophan halogenase AetF bound to the non-native tricyclic substrate tryptoline reveals a binding pose analogous to l-tryptophan that directs C6-first halogenation. Product analysis identifies 6,8-dibromotryptoline as the final product.

## 1. Introduction

Flavin-dependent halogenases (FDHs) mediate the regio­selective halogenation of aromatic and heteroaromatic substrates, thereby generating key building blocks for natural products, pharmaceuticals and agrochemicals (Neumann *et al.*, 2008 ▸; Weichold *et al.*, 2016 ▸; Agarwal *et al.*, 2017 ▸; Phintha *et al.*, 2020 ▸; Gkotsi *et al.*, 2018 ▸; Latham *et al.*, 2018 ▸). Thanks to the mild reaction conditions required and their high regioselectivity, FDHs are a promising alternative to chemical synthesis (Smith & El-Hiti, 2005 ▸). In most known FDHs, halogenation is performed by two-component systems, in which the halogenase relies on a separate flavin reductase to regenerate FADH_2_ (Dong *et al.*, 2005 ▸; Dachwitz *et al.*, 2021 ▸). In contrast, AetF represents a rare single-component tryptophan halogenase: a flavoprotein that combines both reductase and halogenase activity in a continuous polypeptide chain (Adak *et al.*, 2022 ▸). AetF catalyzes a sequential dibromination of tryptophan, first at C5 to form 5-bromotryptophan (5-Br-Trp) and subsequently at C7 to yield 5,7-dibromotryptophan (5,7-Br_2_-Trp), and thus initiates the biosynthesis of the cyanobacterial neurotoxin aetokthonotoxin (Breinlinger *et al.*, 2021 ▸).

The first experimental structures of AetF revealed two dinucleotide-binding domains as found in most flavin-containing monooxygenases (FMOs): one tightly binding FAD and the other presumed to bind NADPH. Unlike FMOs, an ∼16 Å tunnel links the FAD binding site to the distant substrate-binding site. Importantly, comparison of the Trp-bound and 5-Br-Trp-bound structures showed an indole flip upon monobromination. The indole of tryptophan binds in an orientation to present C5 for halogenation, while after bromination the flipped pose of tryptophan places C7 in the same productive position for the second halogenation (Gäfe & Niemann, 2023 ▸).

Recent studies have highlighted the exceptional catalytical versatility of AetF. Lewis and coworkers showed that AetF exceeds the catalytic capabilities of FDH variants generated in several rounds of directed evolution and can halogenate a wide variety of aromatic and heterocyclic substrates with high site selectivity and enantioselectivity, including atroposelective and cycloiodoetherification transformations (Jiang *et al.*, 2022 ▸). AetF can be used for selective C—H halogenation of alkenes and alkynes, often with high stereoselectivity (Jiang *et al.*, 2024 ▸), and to site-selectively install two different halogens (bromine or iodine) in tryptophan (Li *et al.*, 2025 ▸; Montua *et al.*, 2025 ▸).

Despite these advances, structural insights into AetF complexes with non-native substrates other than 7-Br-Trp (Gäfe & Niemann, 2023 ▸) are still lacking, and such insights are generally rare among FDHs (Schnepel *et al.*, 2023 ▸; Andorfer *et al.*, 2022 ▸). We recently published a structure of the two-component halogenase Thal in complex with tryptoline (Bork *et al.*, 2025 ▸). The study aimed to rationalize the experimentally observed regioselectivity on a structural level to guide further site-directed mutagenesis and expand the substrate scope of Thal. Since single- and two-component halogenases differ greatly in sequence and structure (Gäfe & Niemann, 2023 ▸), the structures of two-component halogenases are likely not suited to guide adjustments of single-component halogenases. Therefore, we aimed to determine a structure of AetF in complex with tryptoline, a member of the β-carboline family. This class of natural and synthetic indole-containing compounds exhibits diverse biological activities, including anticancer, neuropharmacological and antimalarial effects (Kushwaha *et al.*, 2023 ▸; Ayipo *et al.*, 2021 ▸; Tshikhudo *et al.*, 2024 ▸). Tryptoline can, for example, interact with imidazoline receptors and act as an inhibitor of monoamine oxidase A (Husbands *et al.*, 2001 ▸; Herraiz & Chaparro, 2006 ▸).

Here, we show that AetF converts tryptoline to two different monobrominated intermediate products and one dibrominated final product. The brominated positions in tryptoline correspond to those of the natural substrate l-Trp in the shared indole moiety. Crystal structures of AetF in complex with tryptoline and 8-bromotryptoline reveal the structural basis of regioselective halogenation and suggest two potential pathways to the formation of 6,8-dibromotryptoline.

## 2. Materials and methods

### 2.1. Protein expression and purification

The C-terminally truncated variant AetF_1–655_ was expressed and purified as described in Gäfe & Niemann (2023 ▸).

### 2.2. Crystallization

AetF_1–655_ was diluted to 6 mg ml^−1^ (50 m*M* HEPES pH 7, 100 m*M* NaCl, 1 m*M* DTT) and crystallized at 20°C in MRC 2-lens sitting-drop plates (SWISSCI) using condition A6 [30 m*M* CaCl_2_, 30 m*M* MgCl_2_, 0.1 *M* HEPES–MOPS pH 7.5, 20%(*v*/*v*) ethylene glycol, 10%(*w*/*v*) PEG 8000] from the Morpheus screen (Molecular Dimensions) with a drop ratio of 300 nl protein solution mixed with 300 nl reservoir solution. The complex structure with tryptoline was obtained by drying 300 nl tryptoline (50 m*M* in H_2_O) on the crystallization plate prior to crystallization. Yellow spear-tip-shaped crystals appeared within five days. For the complex with 8-bromotryptoline, 500 nl 50 m*M* isolated monobrominated product from preparative halogenation was dried on an MRC 3-lens sitting-drop plate (SWISSCI). After adding 500 nl reservoir solution to the dried ligand, pre-grown AetF crystals were transferred to the soaking droplet and incubated for 1 h. All crystals were flash-cooled in liquid nitrogen without additional cryoprotectant.

### 2.3. Data collection and structure determination

X-ray diffraction data were collected at the PETRA III storage ring (DESY, Hamburg, Germany) using *MxCuBE* (Gabadinho *et al.*, 2010 ▸; Eguiraun *et al.*, 2016 ▸) at 100 K at λ = 0.9763 Å on beamline P13 (Cianci *et al.*, 2017 ▸) for AetF–tryptoline and at λ = 0.9184 Å on beamline P14 for AetF–8-bromotryptoline. The diffraction data were processed using *XDS* (Kabsch, 2010 ▸). The AetF–tryptoline data were scaled with *STARANISO* (Tickle *et al.*, 2018 ▸) with 〈*I*/σ(*I*)〉 = 1.20 as the cutoff criterion and the resolution limit of the anisotropically truncated data was verified by *PAIREF* (Malý *et al.*, 2020 ▸; Karplus & Diederichs, 2012 ▸). The AetF–8-bromotryptoline data were scaled with *XSCALE* (Kabsch, 2010 ▸). Both structures were solved via rigid-body refinement in *phenix.refine* (Afonine *et al.*, 2012 ▸; Liebschner *et al.*, 2019 ▸) using PDB entry 8cjd as a starting model.

Both models were optimized by several rounds of model building in *Coot* (Emsley *et al.*, 2010 ▸) and refinement in *phenix.refine* (Afonine *et al.*, 2012 ▸; Liebschner *et al.*, 2019 ▸) using TLS parameters (generated by *phenix.refine*), optimizing both X-ray/stereochemistry and X-ray/ADP weights, and automatic addition of hydrogens. For the AetF–tryptoline structure chain *A* of PDB entry 8cjd was used for reference-model restraints and the twin law −*k*, −*h*, −*l* (twin fraction 0.290) was determined by twin refinement in *REFMAC*5 (Kovalevskiy *et al.*, 2018 ▸) and used for all refinements. Data-collection and refinement statistics are summarized in Tables 1 ▸ and 2 ▸, respectively. Coordinates and structure factors of the final model were deposited in the Protein Data Bank with accession codes 9h6z (AetF–tryptoline) and 29oi (AetF–8-bromotryptoline). All structural illustrations were generated in *PyMOL*.

### 2.4. Expression, purification and activity determination of phosphite dehydrogenase (PTDH)

Expression, purification and activity determination of His-PTDH were performed as described in Bork *et al.* (2025 ▸).

### 2.5. Analytical tryptoline conversion

Analytical halogenation assays were performed using tryptoline as the substrate and AetF as the halogenase. Standard reactions (100 µl) contained 1 m*M* tryptoline, 25 µ*M* AetF, 15 m*M* sodium phosphate buffer pH 7.4, 30 m*M* NaBr or NaI, 50 m*M* Na_2_HPO_3_, 50 µ*M* FAD, 1 m*M* NADP^+^, 0.5 µ*M* EDTA and 50 U ml^−1^ catalase from bovine liver (Sigma–Aldrich). Phosphite dehydrogenase (2 U ml^−1^, PTDH) was included as an NADPH-regeneration system. Reactions were initiated by addition of AetF and incubated at 25°C with gentle agitation (500 rev min^−1^). After 4 h or overnight incubation, reactions were quenched by adding an equal volume of acetonitrile. Samples were centrifuged (30 min, 30 000*g*, 4°C) and the clarified supernatant was analyzed by RP-HPLC.

### 2.6. Preparative tryptoline conversion

To determine the regioselectivity during the halogenation of tryptoline, a reaction was set up similarly to as described in Section 2.5, with 2 m*M* tryptoline, a reaction volume of 25 ml and NaBr as a halide source. The reaction was performed in an Erlenmeyer flask with gentle shaking for oxygen supply at 25°C. Reaction progress was monitored by analytical RP-HPLC measurements. To ensure an adequate yield of both brominated products, half of the reaction was stopped after 5 h of incubation with acetonitrile (1:1), while the second half was stopped after incubation overnight. The precipitated proteins were removed by centrifugation, and the soluble supernatants from both halves were combined and lyophilized overnight. The product was resolved in water:acetonitrile (1:1) and purified by preparative RP-HPLC. LC-MS-verified fractions containing monobrominated or dibrominated tryptoline were pooled separately and lyophilized. For NMR analysis the halogenated products were dissolved in DMSO-d_6_. NMR spectra were acquired using a Bruker Avance NEO 600 spectrometer (^1^H, 600 MHz; ^1^^3^C, 151 MHz). Chemical shifts δ (p.p.m.) are reported relative to the residual solvent signal of DMSO-d_6_ (^1^H, 2.50 p.p.m.; ^13^C, 39.5 p.p.m.).

### 2.7. Reversed-phase high-performance liquid chromatography (RP-HPLC)

For analytical measurements, a Shimadzu Nexera XR chromatography system was used, equipped with a diode-array detector (SPD-M20A IVDD), pump (Nexera XR LC-20AD), column oven (CTO-20A) and autosampler (Nexera XR SIL-20A). Separation was carried out on a Luna 3 µm C_18_ column (Phenomenex, 100 Å, 100 × 2 mm) at 0.65 ml min^−1^. UV absorbance was monitored at 220, 254 and 280 nm. The eluents were (A) water with 0.1% trifluoroacetic acid (TFA) and (B) acetonitrile with 0.1% TFA. The column was maintained at 40°C and elution was performed with the following gradient: 5–95% B over 0–2.5 min, 95% B from 2.5 to 3.2 min, 95–5% B from 3.2 to 3.21 min and 5% B from 3.21 to 5.0 min.

Preparative RP-HPLC was performed on a Shimadzu Nexera system using a Luna 5 µm C_18_ column (Phenomenex, 100 Å, 250 × 21.2 mm) at 10 ml min^−1^. The eluents were (A) 95% water/5% acetonitrile with 0.1% trifluoroacetic acid (TFA) and (B) 95% acetonitrile/5% water with 0.1% TFA. Elution was performed with the following gradient: 0% B over 0–10 min and 0–90% B from 10 to 70 min.

### 2.8. High-performance liquid chromatography–mass spectrometry (LC-MS)

Analytical HPLC-MS measurements were performed on a Shimadzu 2040 HPLC system coupled to a Shimadzu LCMS 2050 mass spectrometer, equipped with a heated DUIS (dual ion ESI/APCI) source, an autosampler, degasser, quaternary low-pressure gradient pump, column oven and photodiode-array detector. Separation was carried out on a Kinetex C_18_ column (Phenomenex, 2.6 µm, 100 Å, 2.1 × 100 mm) at 0.45 ml min^−1^ and 40°C column oven temperature. The DUIS source was operated at 450°C with a desolvation line temperature of 200°C, using 3.0 kV for positive ionization and −2.0 kV for negative ionization. Nitrogen from an NGA CASTORE XS iQ 18 generator was used as a nebulizer (2 l min^−1^), drying (5 l min^−1^) and heating (7 l min^−1^) gas. The mobile phase consisted of A (H_2_O/acetonitrile/formic acid, 950:50:1) and B (acetonitrile/H_2_O/formic acid, 950:50:1) with a linear gradient: 0% B from 0 to 0.5 min, 0–100% B from 0.5 to 5.5 min, 100% B from 5.5 to 6.0 min, 100–0% B from 6.0 to 6.01 min and 0% B from 6.01 to 10.11 min.

### 2.9. Accurate mass determination

Accurate mass nano-electrospray ionization (ESI) measurements were performed using a Synapt G2Si quadrupole ion mobility spectrometry time-of-flight (Q-IMS-TOF) mass spectrometer (Waters GmbH, Manchester, UK) in resolution mode, interfaced to a nano-ESI ion source. Nitrogen served both as the nebulizer gas and the dry gas and was generated by an NGM 11 nitrogen generator. Samples were dissolved in acetonitrile and introduced by static nano-ESI using in-house pulled glass emitters. The mass axis was internally calibrated with the protonated leucine enkephalin ion as a calibration standard.

## 3. Results and discussion

### 3.1. AetF converts tryptoline mainly to 6-bromotryptoline and 6,8-dibromotryptoline

**Figure 1:**
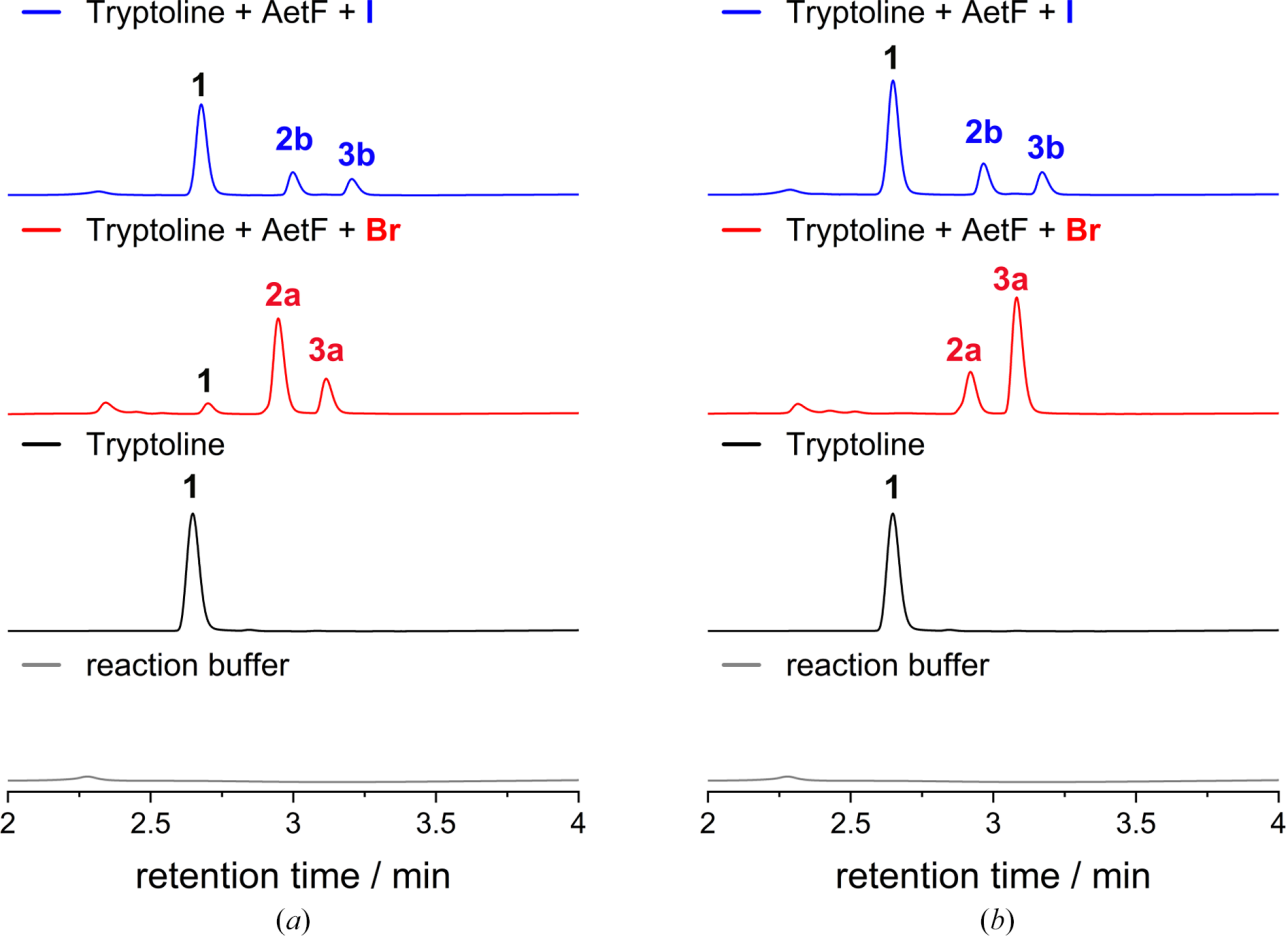
Conversion of tryptoline catalyzed by AetF. Analytic RP-HPLC chromatograms recorded at 280 nm show the conversion of tryptoline by AetF in the presence of bromide (red) or iodide (blue) after (*a*) 4 h and (*b*) overnight reaction. In both the bromination and iodination assays, two distinct products occur, identified by LC-MS as monohalogenated (**2a**, **2b**) and dihalogenated (**3a**, **3b**) derivatives of tryptoline.

**Figure 2:**
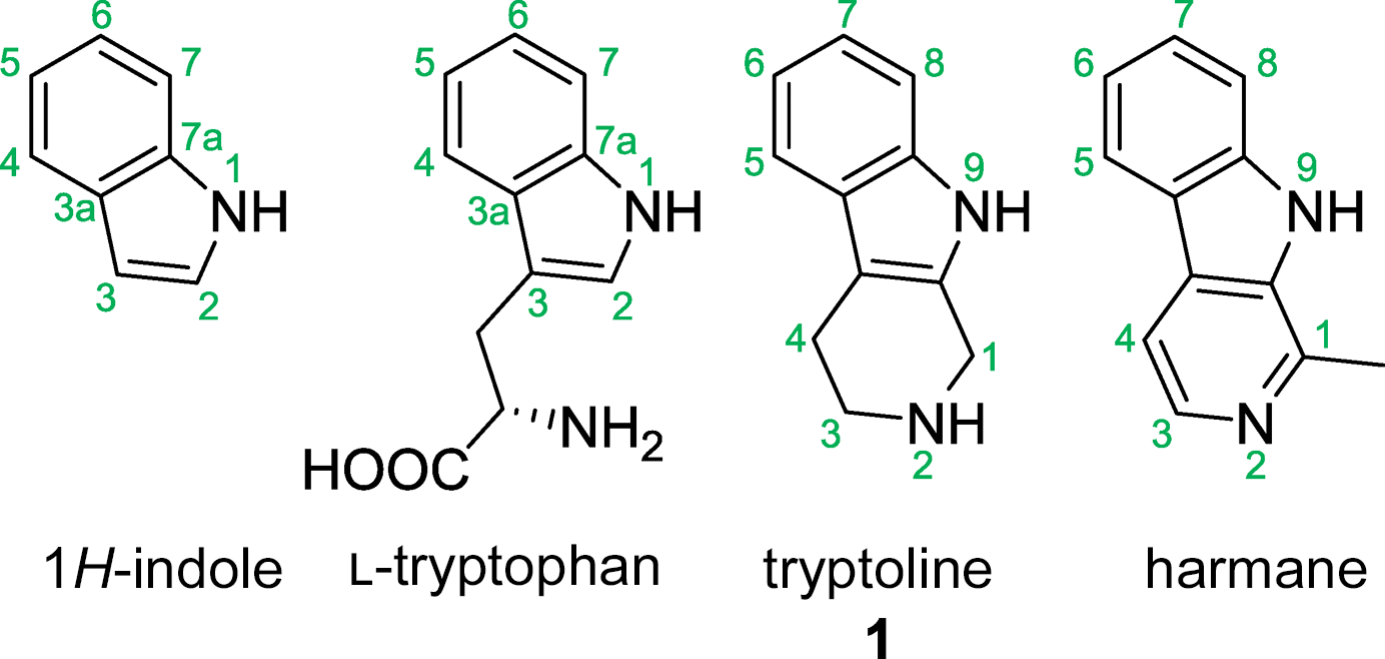
Numbering of various indole-containing compounds for better comparison of regioselectivity.

To confirm the β-carboline tryptoline (**1**) as a substrate of AetF, bromination and iodination were examined in enzymatic assays and analyzed by RP-HPLC (Fig. 1 ▸). In both cases, two products occurred after 4 h, corresponding to monohalogenated (**2a**, **2b**) and dihalogenated (**3a**, **3b**) derivatives of tryptoline, in LC-MS analysis (Supplementary Figs. S1 and S2). Extended reaction times (overnight) resulted in further conversion to the dibrominated product (**3a**). However, significant remaining monobrominated tryptoline indicates that the second halogenation step takes more time. In comparison, the conversion in iodination assays was lower at both time points. To determine the regioselectivity, enzymatic bromination of tryptoline by AetF was performed on a preparative scale. The reaction was stopped at a time point where both products occurred simultaneously. The monohalogenated and dihalogenated products were separated by preparative RP-HPLC and analyzed by NMR spectroscopy. The NMR spectra (Supplementary Figs. S3–S12) identified peak **3a** as the pure dihalogenated product 6,8-dibromotryptoline, while peak **2a** consisted of a mixture of monohalogenated products, containing 6-bromotryptoline as the major component and 8-bromotryptoline as a minor side product (approximately 9% according to ^1^H NMR). In tryptoline, indole positions C6 and C8 correspond to C5 and C7 in the native AetF substrate l-Trp (Fig. 2 ▸). Accordingly, AetF dihalogenates tryptoline at the same indole positions as are halogenated in l-Trp. The occurrence of the two different monobrominated products suggests two possible poses of tryptoline bound to AetF, with either the C6 or C8 position oriented towards the catalytic amino acids.

### 3.2. Structural basis for the preferred formation of 6-bromotryptoline

**Figure 3:**
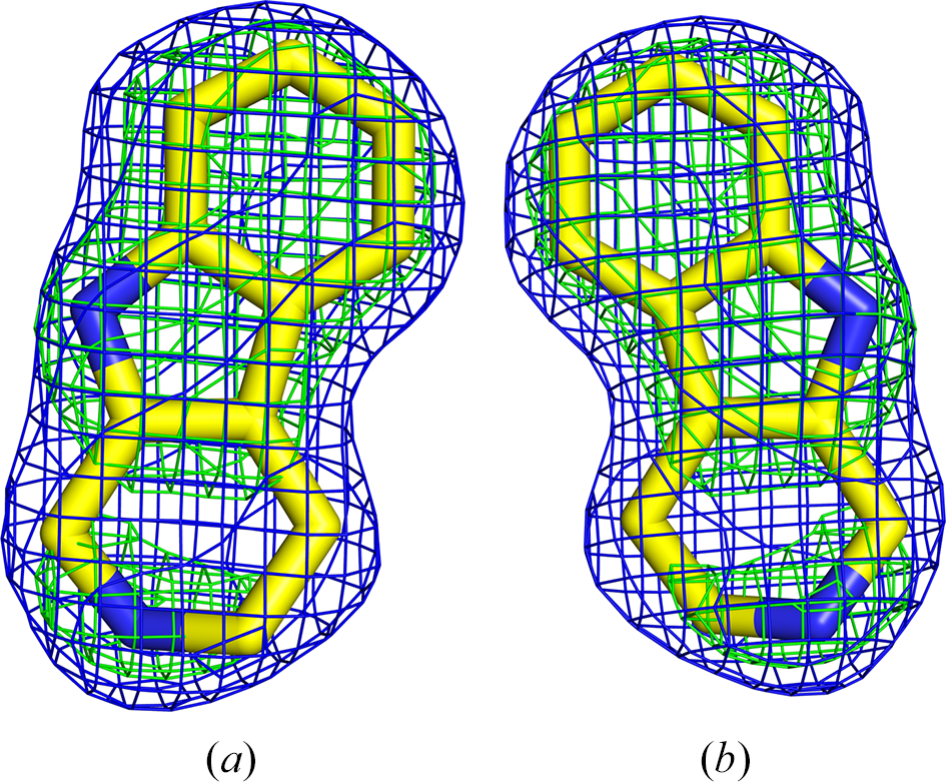
Initial *mF*_o_ − *DF*_c_ (green mesh, +3σ) and final *2 mF*_o_ − *DF*_c_ density (blue mesh, 1.5σ) of tryptoline (yellow C atoms) in chain *A*. The views in (*a*) and (*b*) are rotated by 180°.

To explain the observed regioselectivity and to gain structural insights into the binding of non-native substrates by the single-component halogenase AetF, we co-crystallized tryptoline with AetF using the C-terminally truncated variant AetF_1–655_ which had proved to crystallize readily (Gäfe & Niemann, 2023 ▸). The resulting structure was solved in space group *P*4_3_ via Fourier synthesis with two crystallographically independent chains per asymmetric unit at a resolution of 1.87 Å. The initial model showed easily interpretable difference density in the active center, and after the placement of the ligand and several rounds of refinement the tryptoline was completely enveloped in 2*mF*_o_ − *DF*_c_ (1.5σ) density (Fig. 3 ▸). Occupancies for tryptoline in both chains were determined using occupancy refinement and resulted in values of 0.95 in chain *A* and 0.89 in chain *B*.

**Figure 4:**
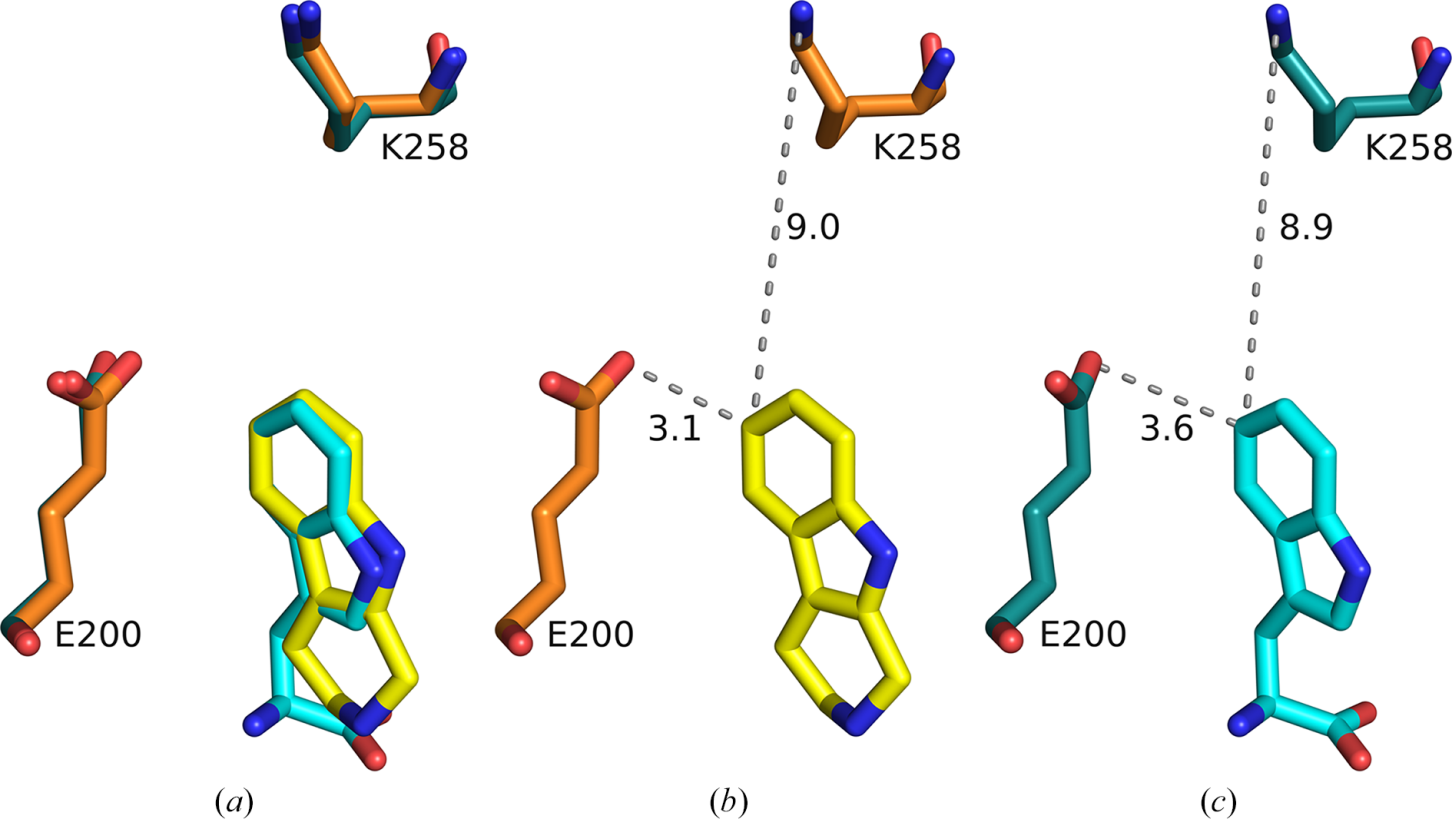
Comparison of the binding poses of tryptoline (yellow C atoms) and the natural AetF substrate l-Trp (cyan C atoms). (*a*) Superposition of AetF–tryptoline (chain *A*) and AetF–l-Trp (PDB entry 8cje, chain *C*) with the catalytic amino acids Lys258 and Glu200 shown (orange C atoms for AetF–tryptoline, dark cyan C atoms for AetF–l-Trp). (*b*, *c*) Distances of the tryptoline C6 atom (*b*) and the l-Trp C5 atom (*c*) to the catalytic amino acids of the respective structure are given in Å.

The binding mode of the tryptoline within the active center results in the indole moiety being almost congruent with the indole of the natural AetF substrate l-Trp (Fig. 4 ▸*a*). This results in similar distances to the catalytic amino acids Lys258 and Glu200 for the C6 of tryptoline (9.0 Å to Lys258 and 3.1 Å to Glu200), shown in Fig. 4 ▸(*b*), as for the catalytically preferred C5 of l-Trp (8.9 Å to Lys258 and 3.6 Å to Glu200), shown in Fig. 4 ▸(*c*). It is important to note that the C6 of tryptoline and C5 of l-Trp share the same position within the indole moiety (Fig. 2 ▸).

**Figure 5:**
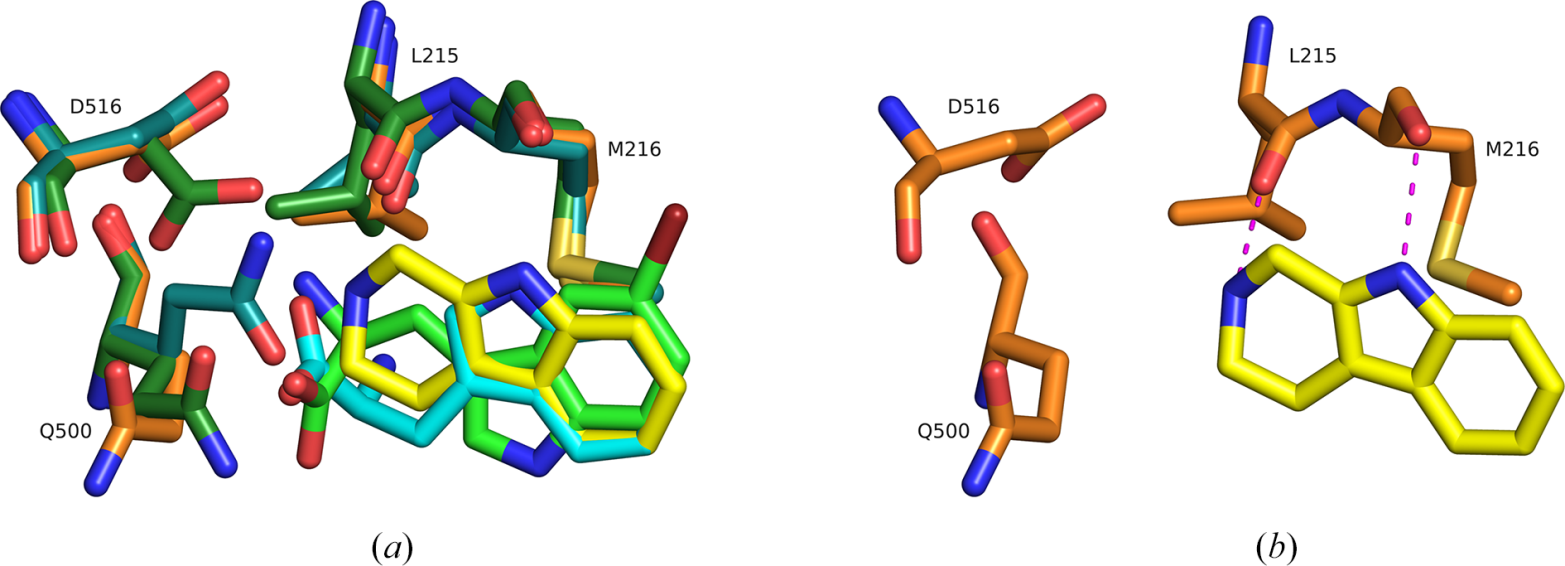
(*a*) Superposition of AetF–tryptoline (chain *A*), AetF–l-Trp (PDB entry 8cje, chain *C*) and AetF–5-Br-Trp (PDB entry 8cjf, chain *A*) with selected amino acids shown (same color coding as in Fig. 4 ▸; dark green C atoms for AetF–5-Br-Trp, with 5-Br-Trp shown in lighter green). Leu215 and Met216 form polar contacts to tryptoline. Gln500 and Asp516 were found to change their conformation upon substrate binding (Gäfe & Niemann, 2023 ▸). (*b*) Polar contacts of tryptoline are shown as magenta dotted lines.

Tryptoline forms two hydrogen bonds to the main-chain O atoms of Leu215 and Met216 (Fig. 5 ▸). These polar contacts in combination with the interactions with surrounding hydrophobic and aromatic amino acids (*e.g.* Leu196, Leu199, Pro219, Phe372 and Phe524) might be crucial to position tryptoline within the active center. For the natural substrates l-Trp and 5-Br-Trp the amino acids Gln500 and Asp516 were found to undergo conformational changes upon substrate binding (Gäfe & Niemann, 2023 ▸). Interestingly, the conformations of these amino acids in the tryptoline-bound structure differ from those of both natural substrates. Mutating these positions might be interesting to enhance the capability of AetF to halogenate β-carbolines or even increase the substrate scope towards larger molecules.

While the observed binding position in AetF–tryptoline explains the preferred C6 halogenation, we assume that AetF is capable of binding tryptoline in a second, flipped orientation, potentially coinciding with an alternative conformation of the substrate, which leads to the formation of the observed minor product 8-bromotryptoline. In this flipped orientation C8 is expected to point towards Glu200. This potential minor binding pose is not visible in the AetF–tryptoline crystal structure. In the absence of further data, the small amount of 8-bromotryptoline compared with 6-bromotryptoline suggests that this alternative binding pose may play only a minor role in the halogenation route.

For the structurally closely related substrate harmane (1-methyl-9*H*-pyrido[3,4-*b*]indole), Lewis and coworkers observed monobromination by AetF at C6 (Jiang *et al.*, 2022 ▸). Since the regioselectivity is the same for harmane and the major monobrominated tryptoline (C6 in both cases), we conclude that harmane might bind to AetF in an orientation similar to that of tryptoline. Lewis and coworkers did not report any signs of double halogenation of harmane, suggesting that the additional methyl group in harmane (see Fig. 2 ▸) prevents the binding of this substrate in the correct orientation for halogenation at C8.

### 3.3. The AetF–8-bromotryptoline structure suggests two potential routes for the formation of dibrominated tryptoline

**Figure 6:**
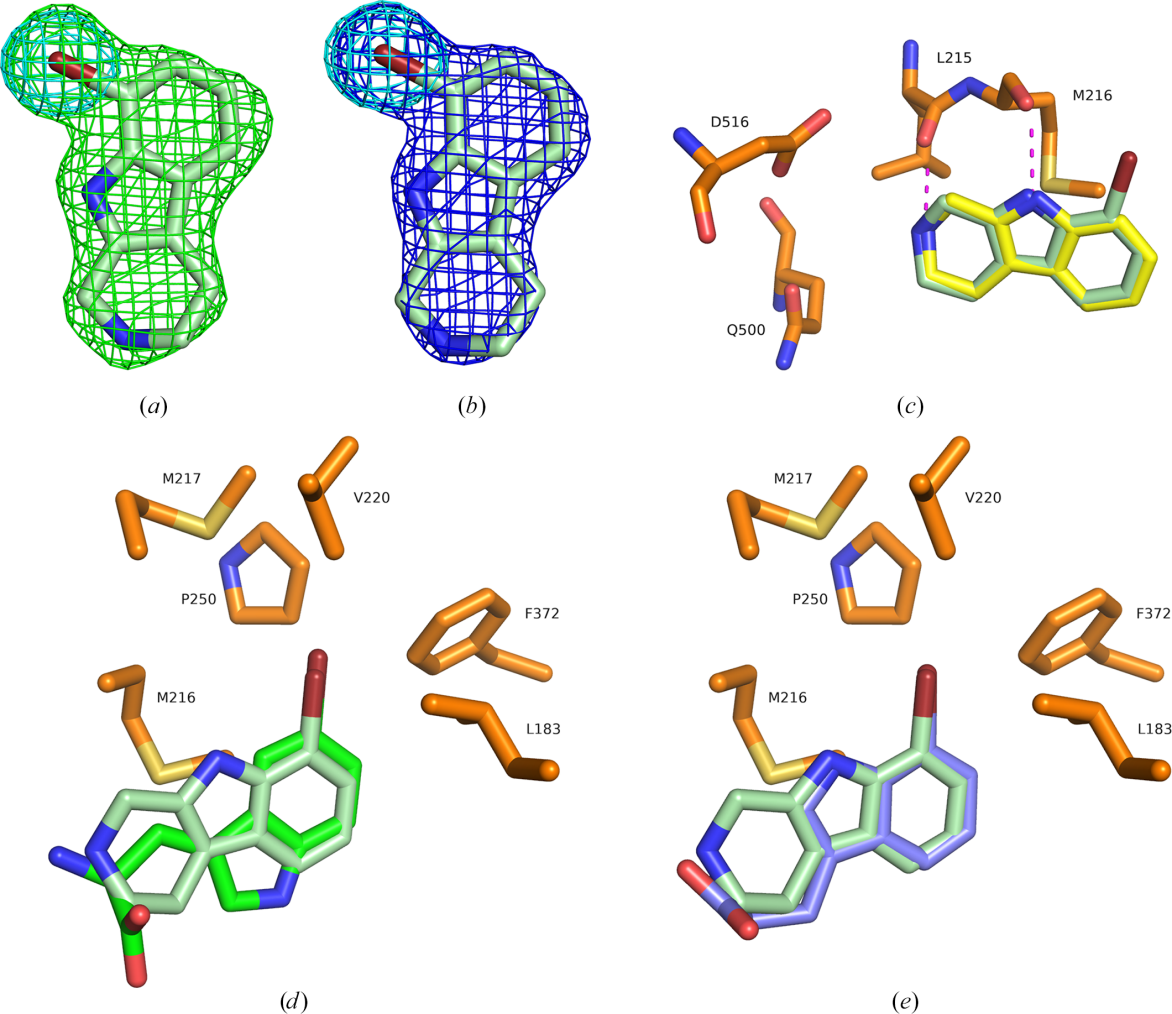
(*a*) Initial *mF*_o_ − *DF*_c_ (green mesh, +3σ) and (*b*) final 2*mF*_o_ − *DF*_c_ density (blue mesh, 1.5σ) of 8-bromotryptoline (pale green C atoms) in chain *A*. Anomalous difference density is shown as as a cyan mesh (5σ). (*c*) Superposition of AetF–8-bromotryptoline (chain *A*) and AetF–tryptoline (chain *A*, yellow C atoms). Polar contacts of 8-bromotryptoline with Leu215 and Met216 are shown as magenta dotted lines. (*d*) Superposition of AetF–8-bromotryptoline (chain *A*) and AetF–5-Br-Trp (chain *A*, green C atoms). (*e*) Superposition of AetF–8-bromotryptoline (chain *A*) and AetF–7-Br-Trp (chain *A*, green C atoms). Selected side chains of the AetF–8-bromotryptoline structure are shown as sticks with orange C atoms in (*c*), (*d*) and (*e*).

We tried to soak both the isolated monohalogenated and dihalogenated products into AetF crystals. The final product 6,8-dibromotryptoline did not bind to AetF crystals under different soaking conditions (∼5–50 m*M*; 1–24 h; 0–10% DMSO). For crystals soaked with the monobrominated products, we surprisingly found 8-bromotryptoline bound to AetF, although 6-bromotryptoline clearly predominates in the NMR spectrum. 8-Bromotryptoline binds in a similar pose as tryptoline (Fig. 6 ▸), with refined occupancies of 0.77 in chain *A* and 0.82 in chain *B*. 8-Bromotryptoline forms the same hydrogen bonds to the main chain of the surrounding amino acids (Leu215 and Met216) as tryptoline, and position C6 faces towards the catalytic glutamate Glu200, potentially allowing efficient C6 halogenation in this binding mode. The bromine substituent inserts into the hydrophobic bromine-binding pocket (Figs. 6 ▸*d* and 6 ▸*e*) known from AetF structures in complex with 5-Br-Trp and 7-Br-Trp (Gäfe & Niemann, 2023 ▸).

The efficient binding of 8-bromotryptoline to the substrate-binding site of AetF in the presence of a large excess of 6-bromotryptoline indicates significantly less stable binding of 6-bromotryptoline. This difference in affinity likely stems from a combination of steric clashes involving the Br atom in the original binding pose and favorable interactions in the flipped orientation of the product. Similar effects had previously been observed for halogenated l-Trp. The monobrominated product 5-Br-Trp binds with a different binding pose to tryptophan, in which a flip of the indole moiety allows bromine to fill a hydrophobic pocket of AetF accompanied by a rearrangement of the backbone atoms of l-Trp and a resulting conformational change of Gln500 of AetF. Bromine in the 8-position of tryptoline fits the bromine-binding pocket of AetF, leaving the binding pose of the tryptoline moiety almost unaltered compared with the nonhalogenated substrate, presumably increasing the binding affinity of 8-bromotryptoline compared with tryptoline. The fact that soaking with 6,8-dibromotryptoline resulted in structures with an empty substrate-binding site suggests that bromine in the 6-position of tryptoline introduces steric conflicts that exceed the presumed attractive contribution of bromine in the 8-position bound to the bromine-binding pocket. Similarly, the flipped binding pose of 5-Br-Trp is presumably caused or supported by steric conflicts of bromine in the 5-position of l-Trp bound to AetF. If halogenated products are overlaid onto the crystallographically observed binding pose of their substrates, the Br atoms in 5-Br-Trp and 6-bromotryptoline and the bromine at the C6 position of 6,8-dibromotryptoline cause clashes with neighboring protein atoms (Supplementary Figs. S13 and S14).

**Figure 7:**
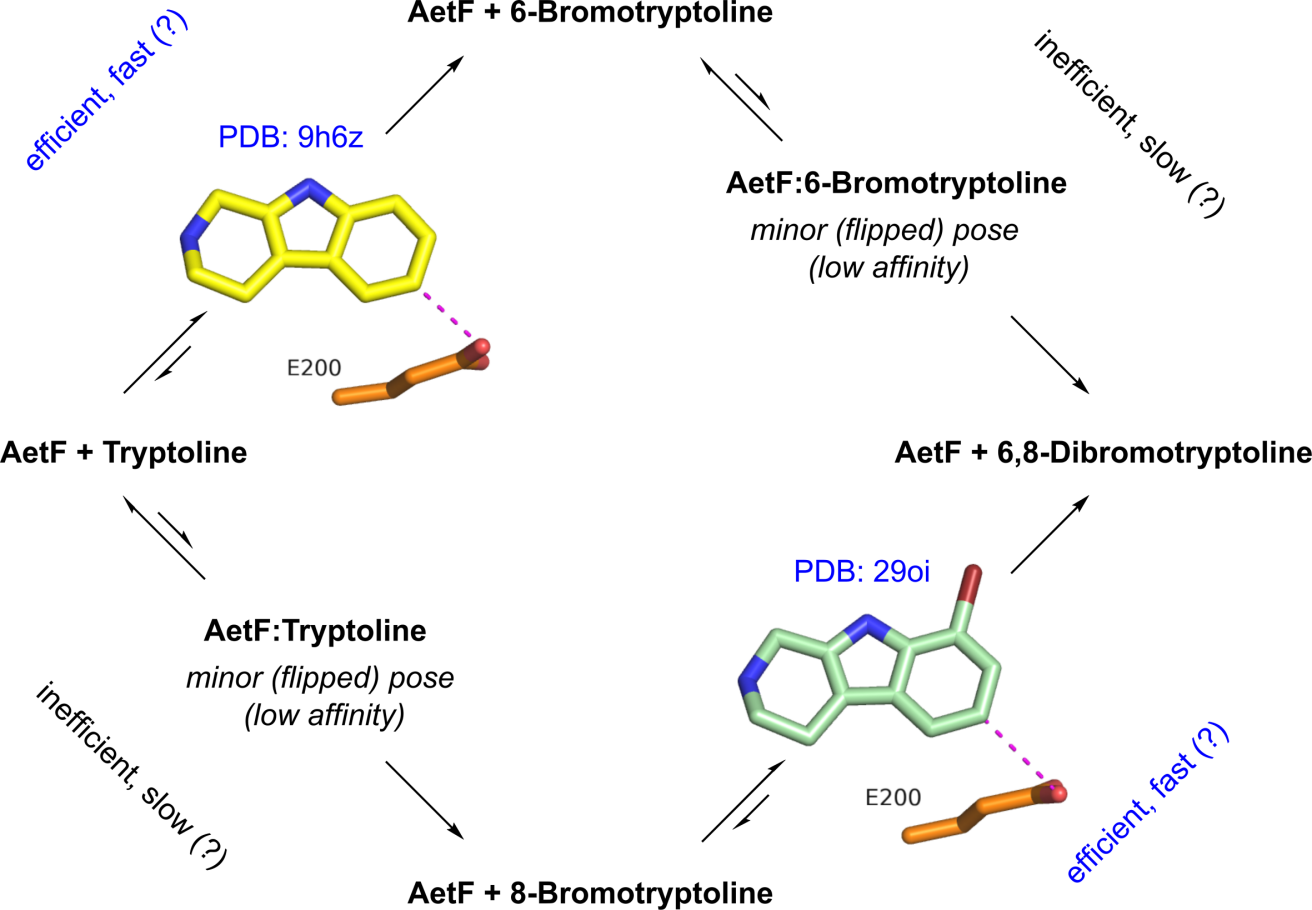
Derived reaction scheme for the double bromination of tryptoline by AetF.

The pronounced difference in binding affinity between the two monobrominated tryptolines produced by AetF suggests that the enzyme exhibits a much lower *K*_m_ value for 8-bromotryptoline than for 6-bromotryptoline. However, the efficiency of the second bromination step also depends on *k*_cat_. Consequently, the catalytic efficiency *k*_cat_/*K*_m_ could be higher for 6-bromotryptoline despite its weaker binding affinity. Hence, the ratio of both monobrominated products in NMR might not correspond to the true ratio of their formation. This complicates the kinetic analysis of individual rate constants (Fig. 7 ▸). However, based on the AetF–tryptoline structure we still expect 6-bromotryptoline as the dominating monobrominated product. This is in agreement with the observation that the second halogenation in the overall reaction appears to be less efficient (Fig. 1 ▸). This is presumably caused by the weaker binding of 6-bromotryptoline in a most likely flipped pose and therefore its less efficient conversion compared with tryptoline and 8-bromotryptoline.

Notably, the initial halogenation of tryptoline into two monohalogenated products by AetF is most likely related to two distinct binding poses of tryptoline in AetF. This differs from the distantly related two-component FDH MalA′ that chlorinates pentacyclic premalbrancheamide in positions 9 and 10 to form dichlorinated malbrancheamide. MalA′ binds its substrate in only one pose that allows almost equally efficient chlorination of positions 9 or 10 corresponding to positions 5 and 6 of the premalbrancheamide indole moiety in the first step (Fraley *et al.*, 2017 ▸).

### 3.4. Nonspecific binding of bromotryptoline near FAD

Interestingly, the AetF–8-bromotryptoline structure also contains an anomalous bromine signal together with near-planar difference density stacked on top of the FAD iso­alloxazine ring in both chains (Supplementary Fig. S15). This hints towards an additional, nonspecific binding position of bromotryptoline. Nonspecific binding of substrate next to the FAD of AetF was previously found for l-Trp, with its indole moiety packed against the isoalloxazine ring (PDB entries 8jz3, 9vwv, 9vwx and 9vwu; Dai *et al.*, 2024 ▸; Li *et al.*, 2025 ▸; Chen *et al.*, 2024 ▸). We modeled both 8-bromotryptoline and 6-bromotryptoline into this density. While the latter explained the density slightly better, we leave this part unmodeled in the deposited structure as the refined density is not sufficiently clear. Also in the AetF–tryptoline structure, a tryptoline binds next to FAD in both chains with reasonable electron density. Relative to the position of bromotryptoline shown in Supplementary Fig. S15, tryptoline is rotated by about 90°, supporting the assumption that binding next to FAD is unspecific.

## 4. Conclusion and outlook

Product analyses using HPLC-MS and NMR spectroscopy combined with two crystal structures suggest that AetF achieves double halogenation of tryptoline by releasing the product of the first halogenation and re-binding it in an alternative orientation before the second halogenation. This reflects what presumably happens during formation of the natural product of AetF, 5,7-Br_2_-Trp (Gäfe & Niemann, 2023 ▸). Previous work successfully shifted the product towards monobrominated tryptophan by mutating AetF and optimizing parameters of the enzymatic tryptophan halogenation reaction (Li *et al.*, 2025 ▸), raising the hope that AetF or designed or evolved AetF variants can be used to produce β-carbolines halogenated at only one specific position, for example pure 6-bromotryptoline or 6-iodotryptoline. Tryptoline and other β-carbolines are widely used as scaffolds in drug design (Ameta, 2024 ▸; Yu *et al.*, 2025 ▸) and bromine or iodine substituents on aromatic carbons facilitate diversification, for example by Suzuki–Miyaura or Buchwald–Hartwig coupling for C—C or C—N bond formation, respectively (Renault *et al.*, 2019 ▸; Dachwitz *et al.*, 2020 ▸). Therefore, enzymatic regioselective monohalogenation of β-carbolines may represent a welcome addition to the toolbox of medicinal chemistry.

## Supplementary Material

PDB reference: AetF–tryptoline, 9h6z

PDB reference: AetF–8-bromotryptoline, 29oi

Supplementary Figures and analysis of brominated products. DOI: 10.1107/S2059798326005954/ag5068sup1.pdf

## Figures and Tables

**Table 1:** Data collection and processing Values in parentheses are for the outer shell.

Structure	AetF–tryptoline	AetF–8-bromotryptoline
PDB code	9h6z	29oi
Diffraction source	P13, DESY	P14, DESY
Wavelength (Å)	0.9763	0.9184
Temperature (K)	100	100
Detector	EIGER X16M	EIGER2 CdTe 16M
Crystal-to-detector distance (mm)	234.175	273.38
Rotation range per image (°)	0.1	0.1
Total rotation range (°)	360	360
Exposure time per image (s)	0.008	0.01
Space group	*P*4_3_	*P*4_3_
*a* = *b*, *c* (Å)	123.05, 87.23	122.60, 86.33
α, β, γ (°)	90, 90, 90	90, 90, 90
Mosaicity (°)	0.115	0.047
Resolution range (Å)	123.05–1.87 (1.96–1.87)	36.9–1.78 (1.89–1.78)
Total No. of reflections	1343748 (64449)	1551970 (247468)
No. of unique reflections	97146 (4768)	121862 (19464)
Completeness, spherical (%)	89.3 (35.0)	99.6 (99.2)
Completeness, ellipsoidal (%)	96.2 (66.5)	—
Multiplicity	13.8 (13.5)	12.7 (12.7)
〈*I*/σ(*I*)〉	12.9 (1.5)	17.2 (1.5)
CC_1/2_	0.997 (0.752)	0.999 (0.686)
*R*_meas_	0.114 (1.840)	0.078 (1.446)
Overall *B* from Wilson plot (Å^2^)	36.22	36.26

**Table 2:** Structure solution and refinement Values in parentheses are for the outer shell.

Dataset	AetF–tryptoline	AetF–8-bromotryptoline
PDB code	9h6z	29oi
Twin law	−*k*, −*h*, −*l*	—
Twin fraction (%)	29	—
Resolution range (Å)	123.05–1.87 (1.90–1.87)	36.9–1.78 (1.80–1.78)
Completeness (%)	90.0 (25.7)	99.65 (98.16)
No. of reflections, working set	91729 (1364)	121862 (3952)
No. of reflections, test set	4962 (73)	5943 (214)
Final *R*_work_ (%)	16.67 (34.47)	16.23 (31.23)
Final *R*_free_ (%)	18.73 (35.06)	18.44 (35.22)
No. of non-H atoms		
Total	11762	11493
Protein	10689	10590
Ligand	318	199
Water	893	704
R.m.s. deviations		
Bond lengths (Å)	0.003	0.003
Angles (°)	0.64	0.64
Average *B* factors (Å^2^)		
Overall	39.78	43.85
Protein	40.01	43.67
Ligand	34.14	44.20
Water	38.10	46.59
Ramachandran plot		
Most favored (%)	98.50	98.02
Allowed (%)	1.50	1.98
Outliers (%)	0.00	0.00
