# Supplementary material for: Structural basis of regioselective double halogenation of the β-carboline tryptoline by the single-component halogenase AetF
Source: Acta Crystallogr D Struct Biol. 2026 Jun 22;82(Pt 7):836–44. doi: 10.1107/S2059798326005954 (PMC13317684; doi:10.1107/S2059798326005954)
Supplement: Supplementary file 1 [file d-82-00836-sup1.pdf]

Acta Cryst D STRUCTURAL BIOLOGY

Volume 82 (2026)

Supporting information for article:

Structural basis of regioselective double halogenation of the β-carboline tryptoline by the single-component halogenase AetF

Simon Bork, Hendrik J. Horstmeier, Bjarne Scharkowski and Hartmut H. Niemann

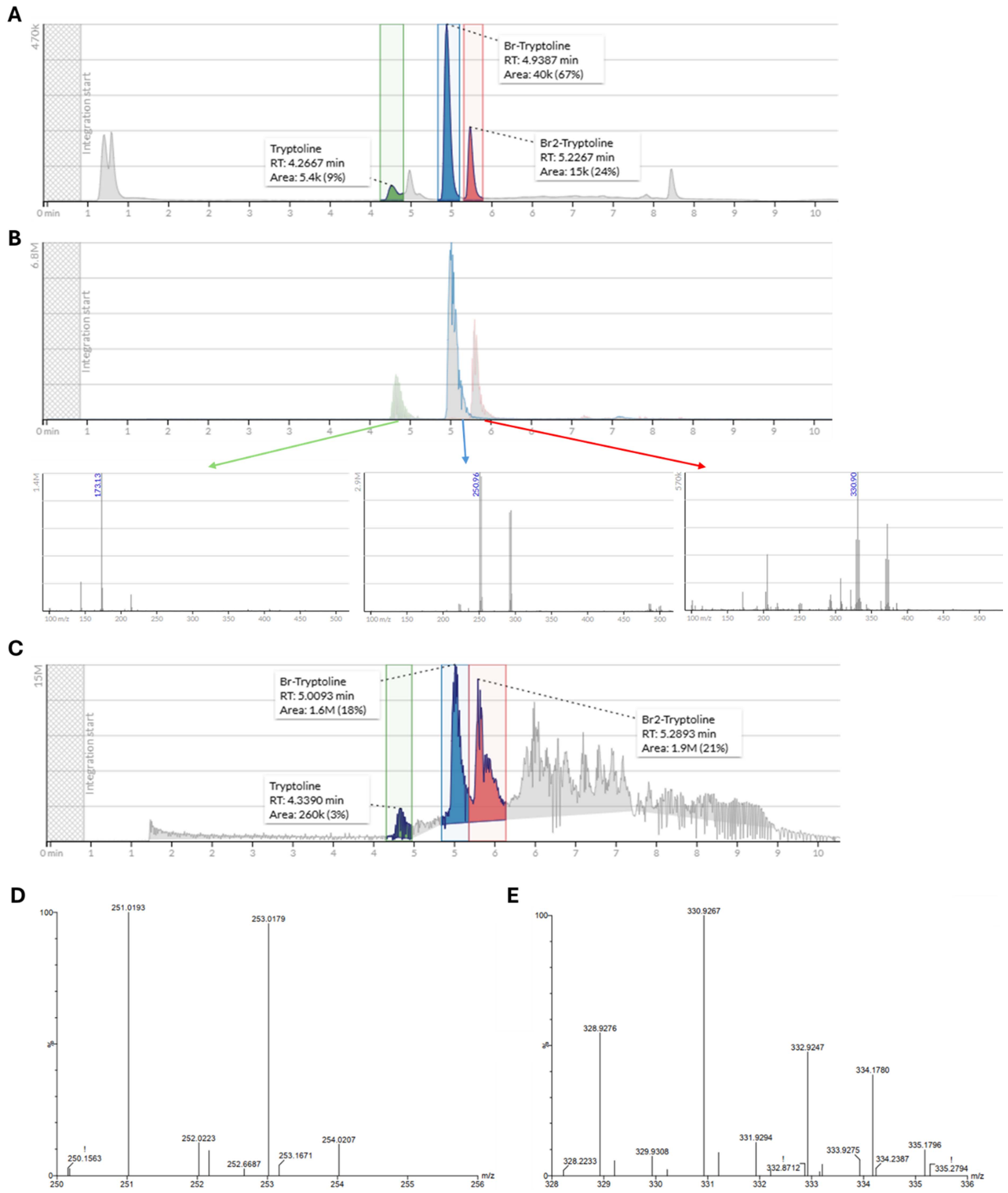

Figure S1 LC-MS analysis for the bromination of tryptoline with AetF. (*a*) UV chromatogram at 280 nm. (*b*) MS-SQD-ES+-Extracted Ion Chromatogram for tryptoline (m/z_{calc}: 173.10; m/z_{exp}: 173.13), Br-tryptoline (m/z_{calc}: 251.13; m/z_{exp}: 250,96) and Br_2-tryptoline (m/z_{calc}: 331.02; m/z_{exp}: 330.90). (*c*) MS-SQD-ES+-Total Ion Chromatogram. (*d*) TOF-MS-ES+ spectrum of Br-tryptoline after preparative RP-HPLC, showing the characteristic isotope ratio of a monobrominated compound. (*e*) TOF-MS-ES+ spectrum of Br_2-tryptoline after preparative RP-HPLC showing the characteristic isotope ratio of a dibrominated compound.

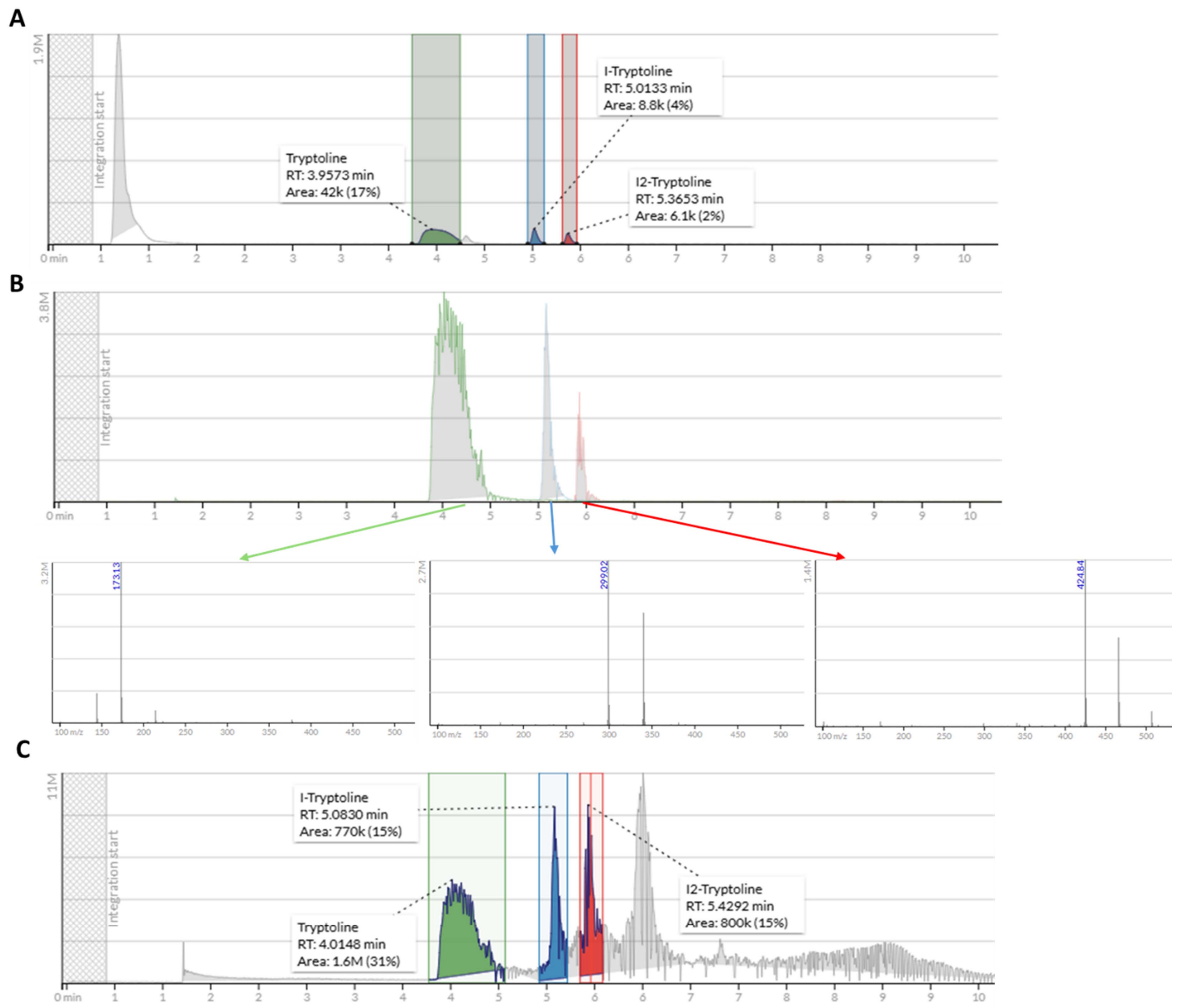

Figure S2 LC-MS analysis for the iodination of tryptoline with AetF. (*a*) UV chromatogram at 280 nm. (*b*) MS-SQD-ES+-Extracted Ion Chromatogram for tryptoline (m/z_{calc}: 173.10; m/z_{exp}: 173.13), I-tryptoline (m/z_{theo}: 299,13; m/z_{exp}: 299,02) and I_2-tryptoline (m/z_{calc}: 424.89; m/z_{exp}: 424.84). (*c*) MS-SQD-ES+-Total Ion Chromatogram.

S1. Analysis of brominated products

S1.1. 6-Bromotryptoline

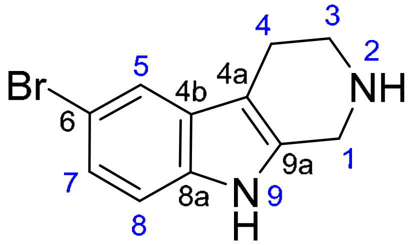

^{1}H NMR (600 MHz, DMSO-d$_6$) δ = 11.28 (s, 1H, N^{9}**H**), 9.22 (s, 1H, N^{2}**H**), 7.68 (d, ^{4}J = 2.1 Hz, 1H, C^{5}**H**), 7.35 (d, ^{3}J = 8.5 Hz, 1H, C^{8}**H**), 7.22 (dd, ^{3}J = 8.6 Hz, ^{4}J = 2.0 Hz, 1H, C^{7}**H**), 4.37 (s, 2H, C^{1}**H$_2$**), 3.45 (t, ^{3}J = 6.1 Hz, 1H, C^{3}**H$_2$**), 2.92 (t, ^{3}J = 6.1 Hz, 2H, C^{4}**H$_2$**).

^{13}C NMR (151 MHz, DMSO-d$_6$) δ = 134.76 (**C^{8a}**), 128.39 (**C^{9a}**), 127.77 (**C^{4b}**), 124.06 (**C^{7}**), 120.36 (**C^{5}**), 113.37 (**C^{8}**), 111.56 (**C^{6}**), 105.37 (**C^{4a}**), 41.48 (**C^{3}**), 40.34 (**C^{1}**), 17.97 (**C^{4}**).

HRMS (ESI+) [M(^{79}Br)+H]$^{+}$ *m/z* 251.01783 (calc.) and *m/z* 251.0193 (exp.).

S1.2. 8-Bromotryptoline

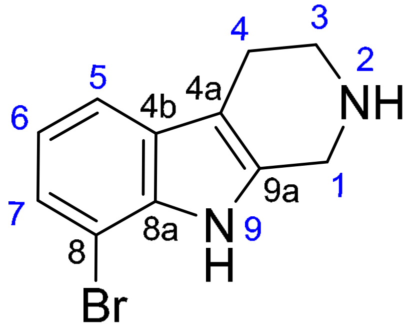

^{1}H NMR (600 MHz, DMSO-d$_6$) δ = 11.45 (s, 1H, N^{9}**H**), 9.22 (s, 1H, N^{2}**H**), 7.39 (d, ^{3}J = 8.17 Hz, 1H, C^{5}**H**), 7.19 (d, ^{3}J = 6.90 Hz, 1H, C^{6}**H**), 7.01 (dd, ^{3}J = 7.86 Hz, 7.86 Hz, 1H, C^{7}**H**), 4.37 (s, 2H, C^{1}**H$_2$**), 3.45 (t, ^{3}J = 6.0 Hz, 1H, C^{3}**H$_2$**), 2.92 (t, ^{3}J = 6.2 Hz, 2H, C^{4}**H$_2$**).

^{13}C NMR (151 MHz, DMSO-d$_6$) δ = 137.12, 128.36, 124.56, 122.81, 122.64, 112.39, 111.11, 105.71, 41.48 (**C^{3}**), 40.34 (**C^{1}**), 17.97 (**C^{4}**).

HRMS (ESI+) [M(^{79}Br)+H]$^{+}$ *m/z* 251.01783 (calc.) and *m/z* 251.0193 (exp.).

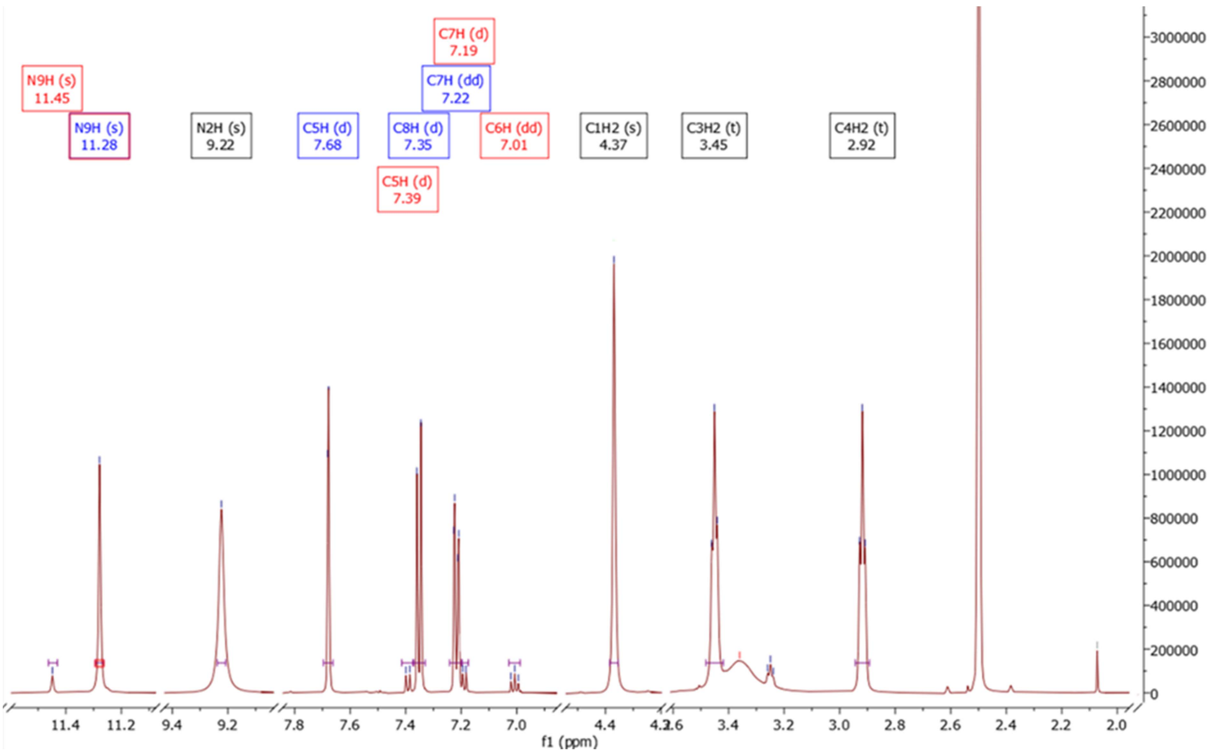

Figure S3 ^{1}H-NMR spectrum of 6-bromotryptoline (blue) and 8-bromotryptoline (red) halogenated with AetF (600 MHz, DMSO-d_6).

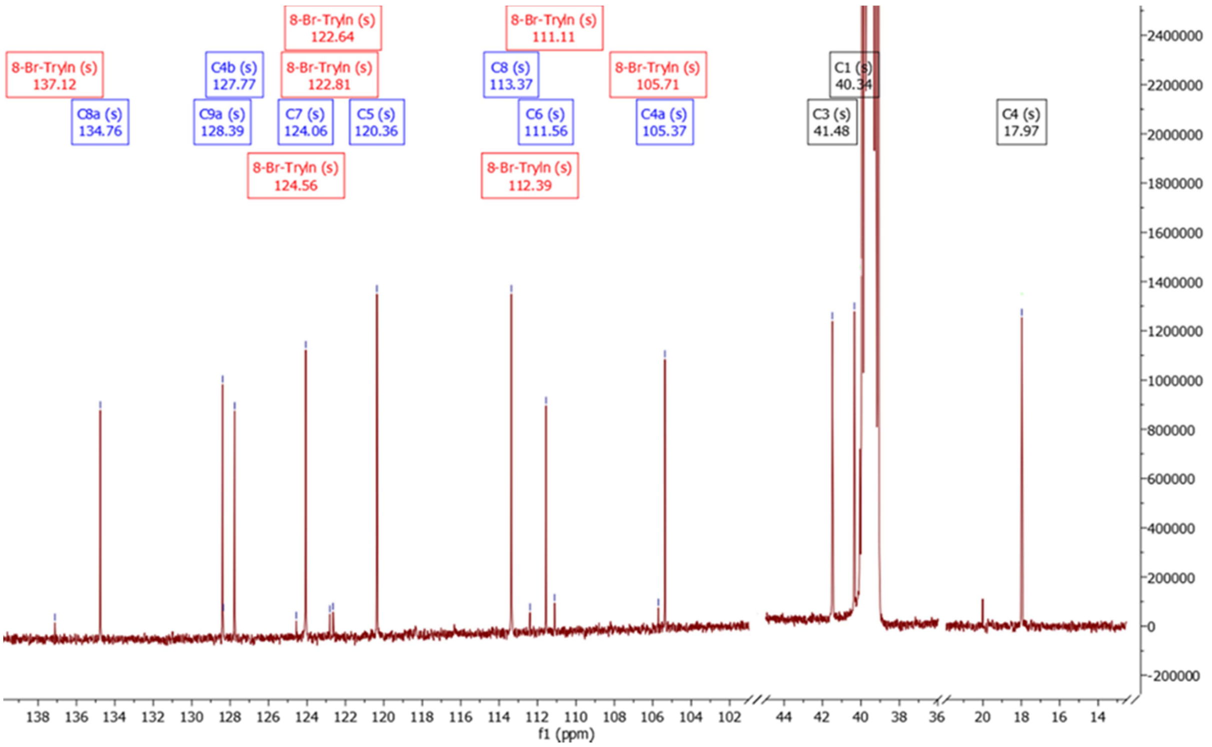

Figure S4 ^{13}C-NMR spectrum of 6-bromotryptoline (blue) and 8-bromotryptoline (red) halogenated with AetF (151 MHz, DMSO-d_6).

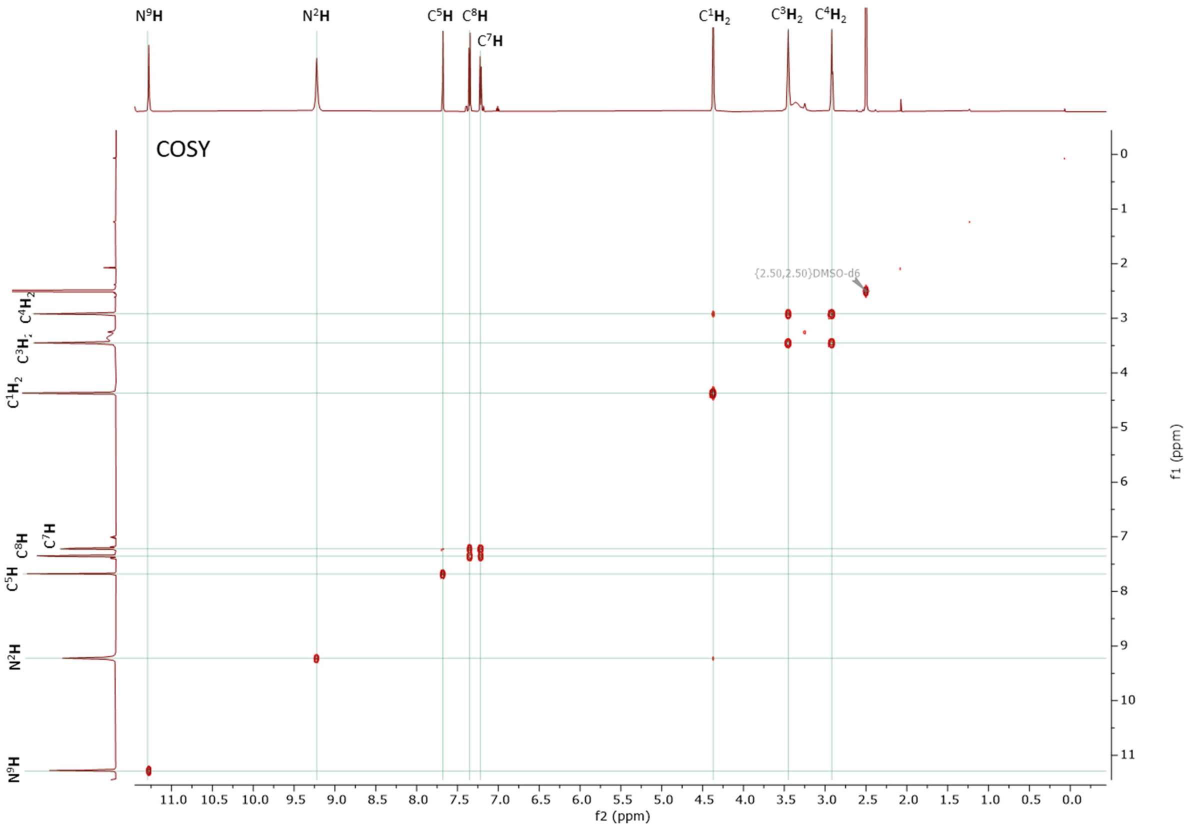

Figure S5 COSY spectrum of 6-bromotryptoline and 8-bromotryptoline halogenated with AetF (600 MHz, DMSO-d_6).

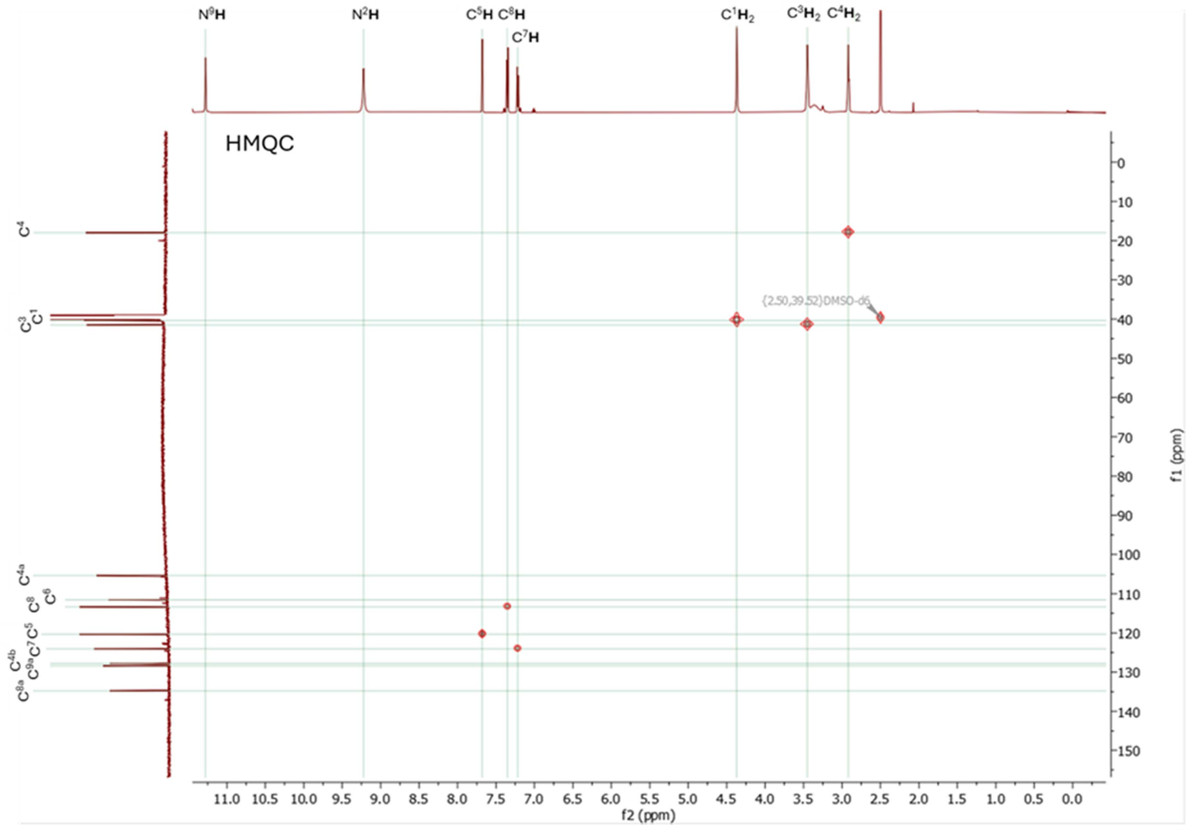

Figure S6 HMQC spectrum of 6-bromotryptoline and 8-bromotryptoline halogenated with AetF (600 MHz, DMSO-d$_6$).

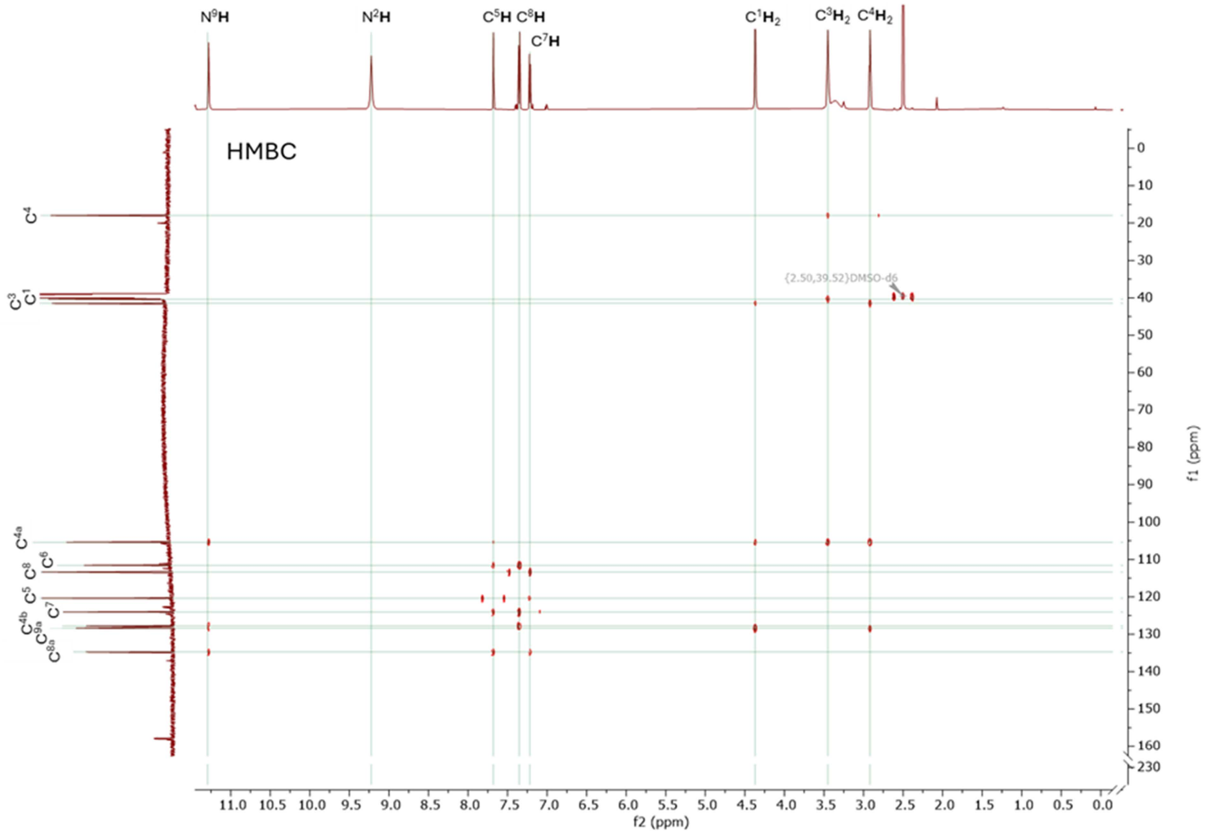

Figure S7 HMBC spectrum of 6-bromotryptoline and 8-bromotryptoline halogenated with AetF (600 MHz, DMSO-d$_6$).

S1.3. 6,8-Dibromotryptoline

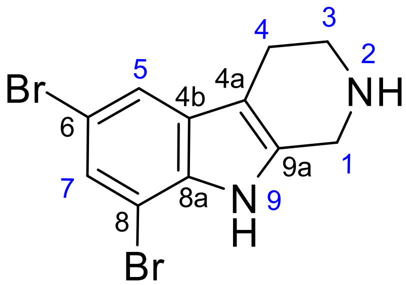

^{1}H NMR (600 MHz, DMSO-d$_6$) δ = 11.50 (s, 1H, N^9**H**), 9.11 (s, 1H, N^2**H**), 7.76 (d, ^4J=1.7, 1H, C^5**H**), 7.51 (d, ^4J=1.7, 1H, C^7**H**), 4.36 (s, 2H, C^1**H$_2$**), 3.45 (t, ^3J=6.0, 1H, C^3**H$_2$**), 2.92 (t, ^3J=6.2, 2H, C^4**H$_2$**).

^{13}C NMR (151 MHz, DMSO-d$_6$) δ = 133.53 (**C^{8a}**), 129.91 (**C^{9a}**), 128.65 (**C^{4b}**), 125.71 (**C^7**), 120.19 (**C^5**), 111.42 (**C^6**), 106.89 (**C^{4a}**), 104.81 (**C^8**), 41.39 (**C^3**), 40.37 (**C^1**), 18.05 (**C^4**).

HRMS (ESI+) [M(^{79}Br)+H]$^+$ *m/z* 328.92834 (calc.) and *m/z* 328.9276 (exp.).

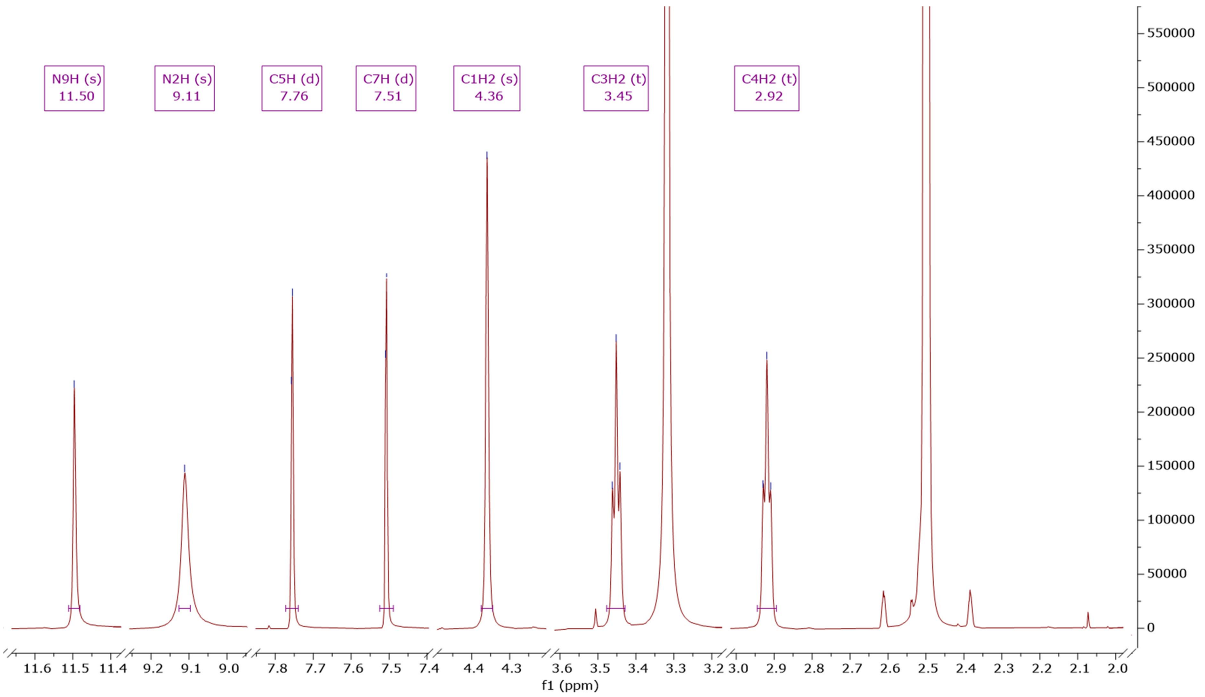

Figure S8 ^{1}H-NMR spectrum of 6,8-dibromotryptoline halogenated with AetF (600 MHz, DMSO-d_6).

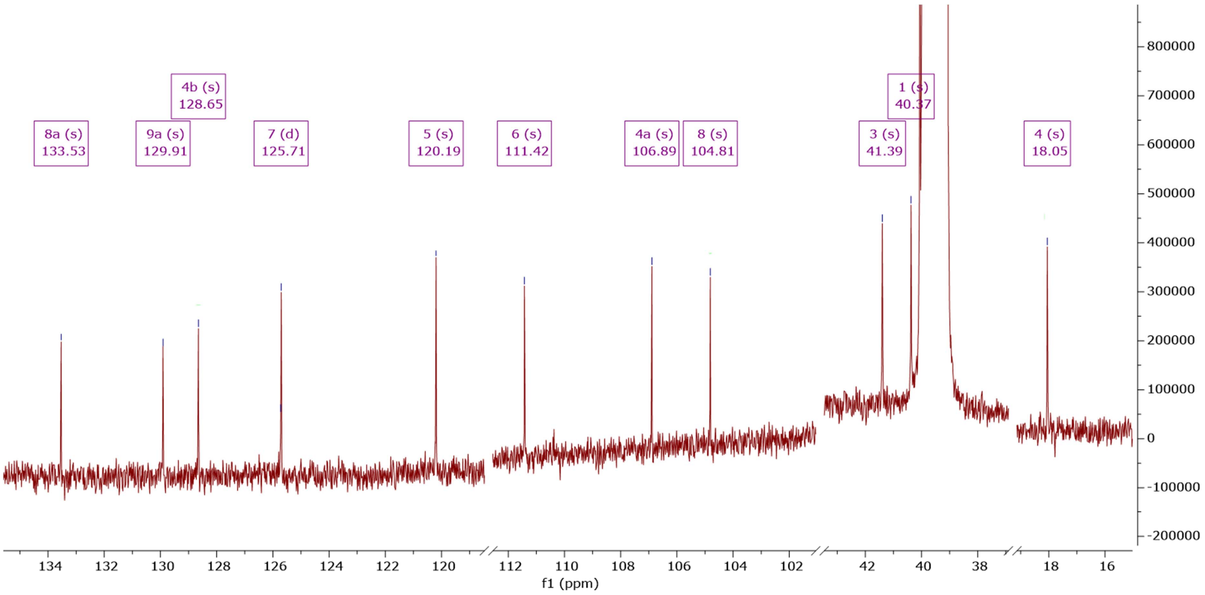

Figure S9 ^{13}C-NMR spectrum of 6,8-dibromotryptoline halogenated with AetF (151 MHz, DMSO-d_6).

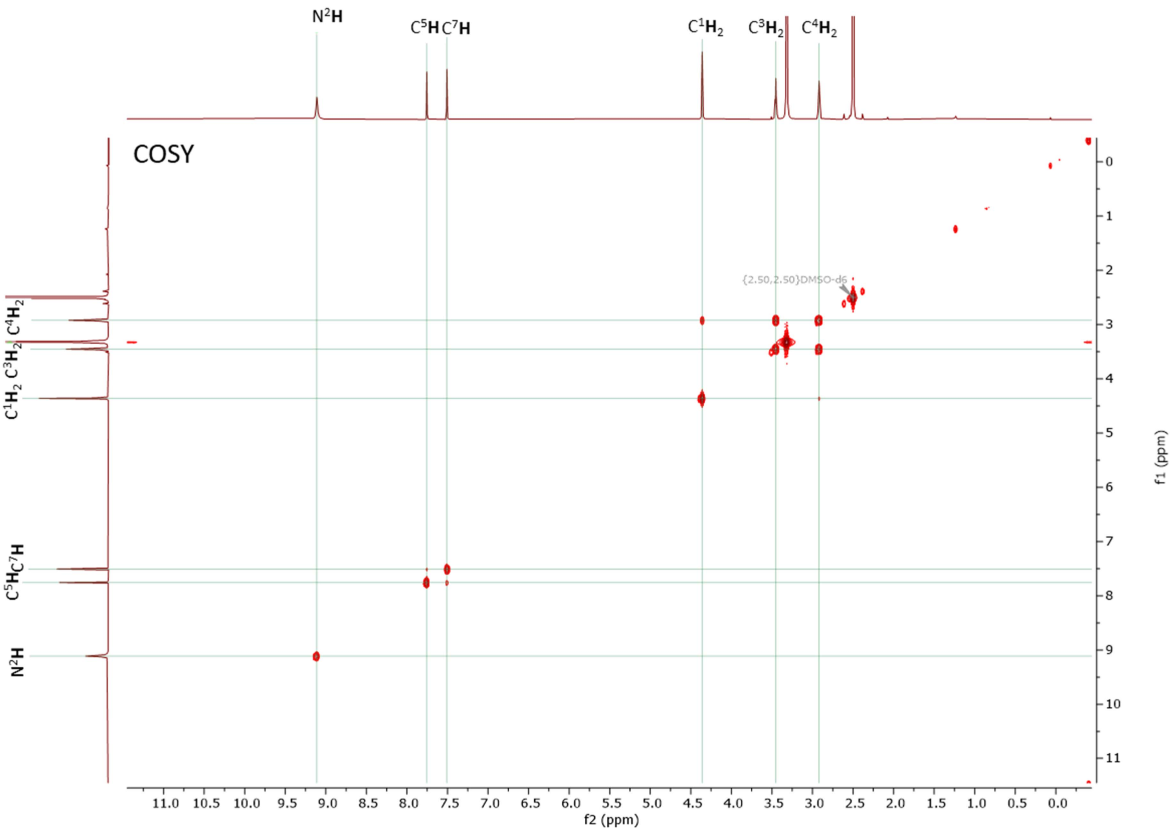

Figure S10 COSY spectrum of 6,8-dibromotryptoline halogenated with AetF (600 MHz, DMSO-d$_6$).

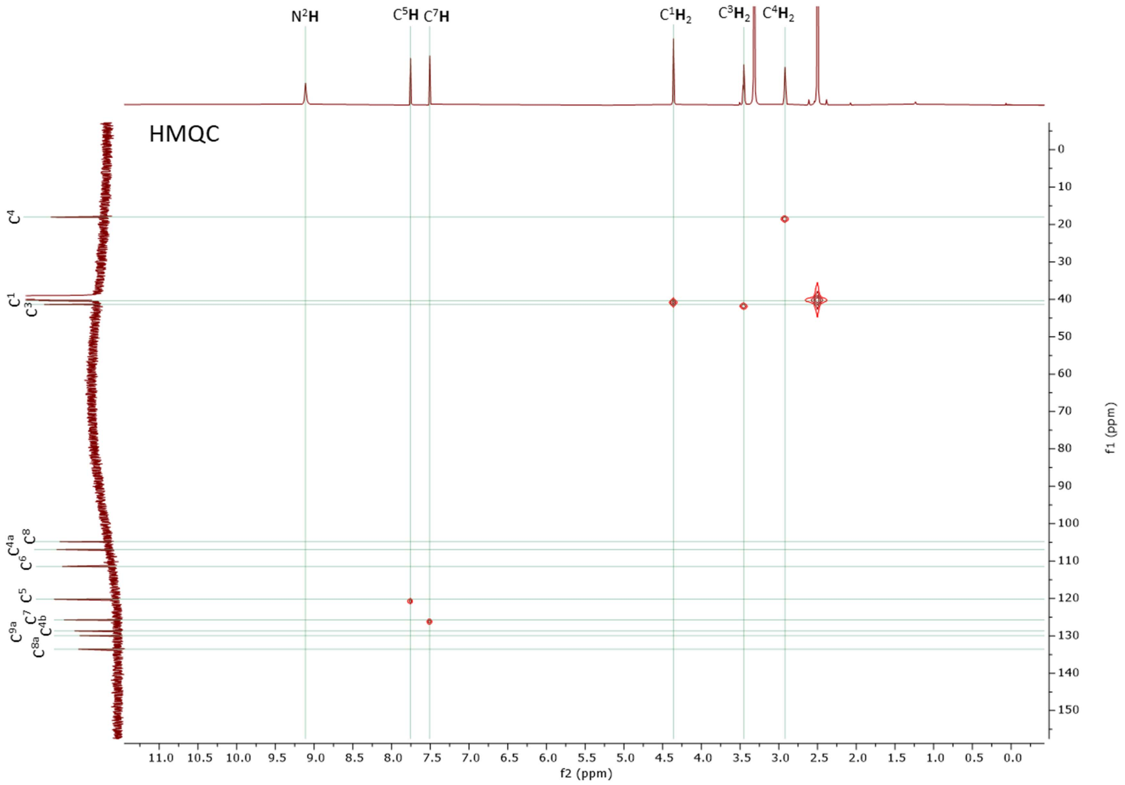

Figure S11 HMQC spectrum of 6,8-dibromotryptoline halogenated with AetF (600 MHz, DMSO-d_6.

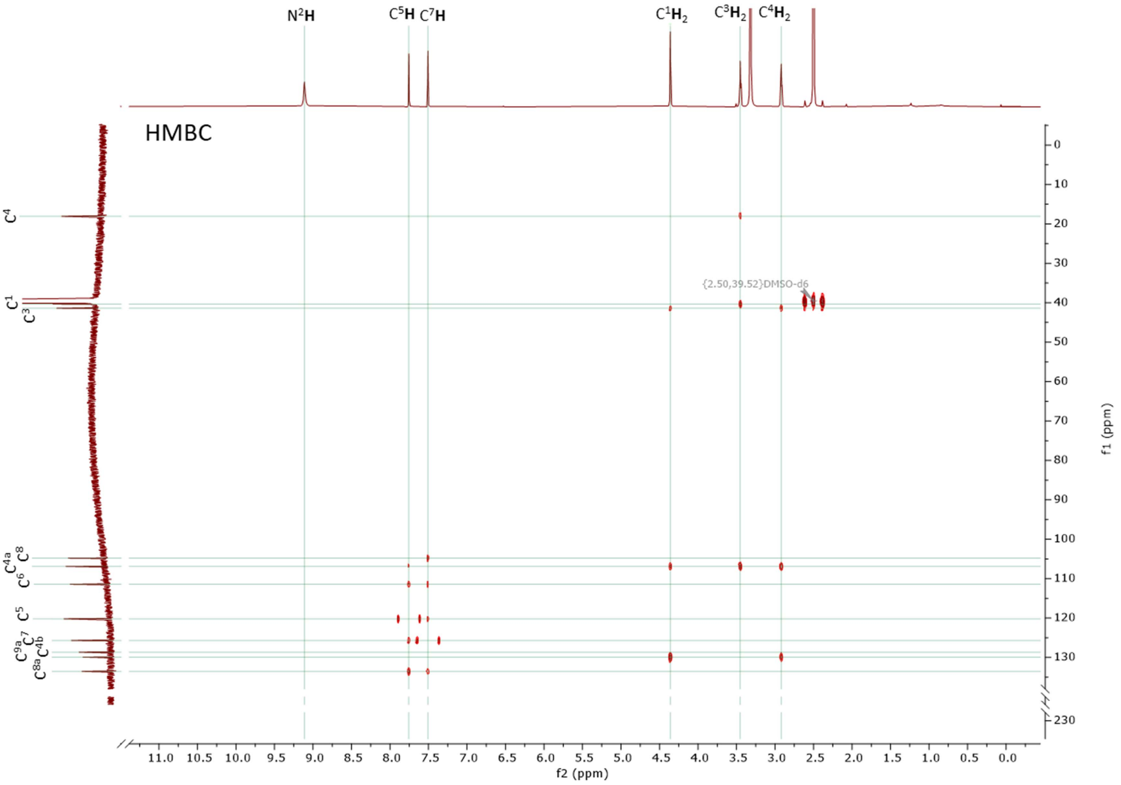

Figure S12 HMBC spectrum of 6,8-dibromotryptoline halogenated with AetF (600 MHz, DMSO-d_6).

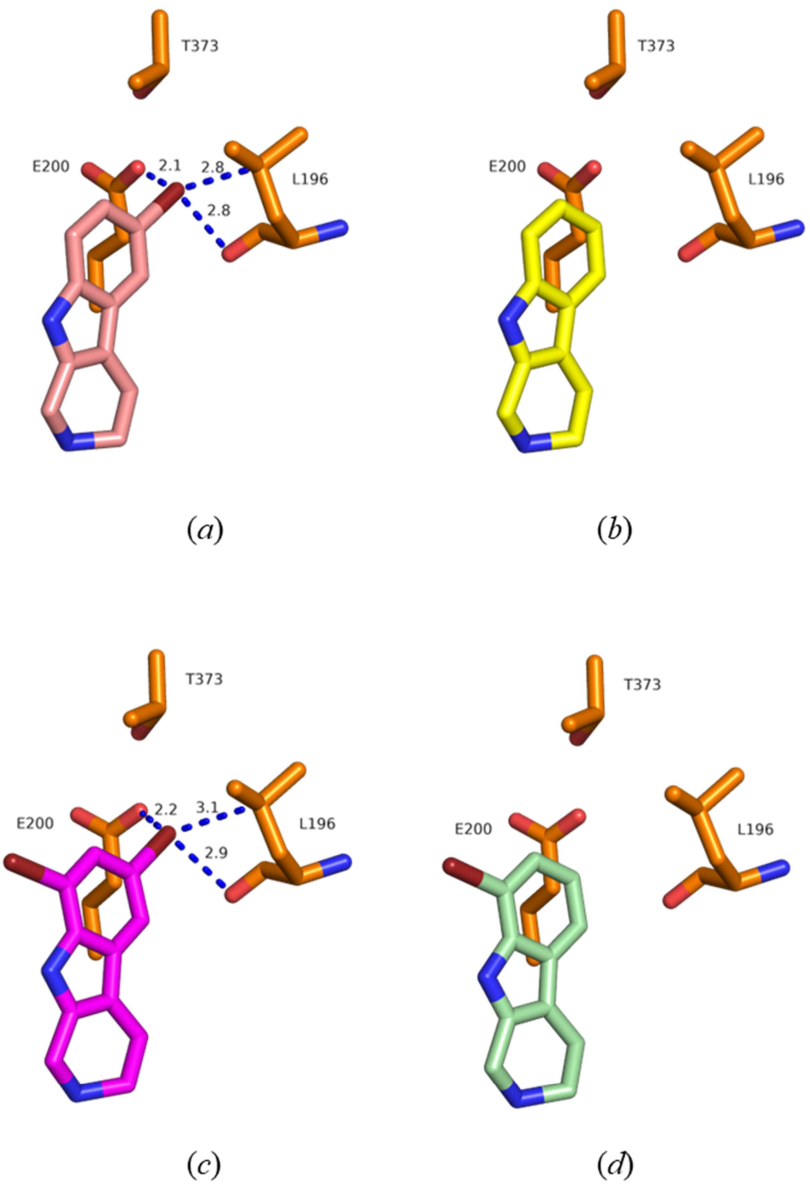

Figure S13 (*a*) 6-Bromotryptoline (salmon carbon atoms) superimposed on tryptoline in AetF–tryptoline. (*b*) Tryptoline (yellow carbon atoms) in AetF–tryptoline (9h6z) as reference. (*c*) 6,8-dibromotryptoline (magenta carbon atoms) superimposed on 8-bromotryptoline in AetF–8-bromotryptoline. (*d*) 8-Bromotryptoline (pale green carbon atoms) in AetF–8-bromotryptoline (29oi) as reference. The blue dashed lines mark clashes of the C6 bromo substituent with surrounding protein atoms. These clashes indicate why 6,8-dibromotryptoline does not bind to AetF in soaking experiments and that 6-bromotryptoline probably has to bind in a flipped orientation.

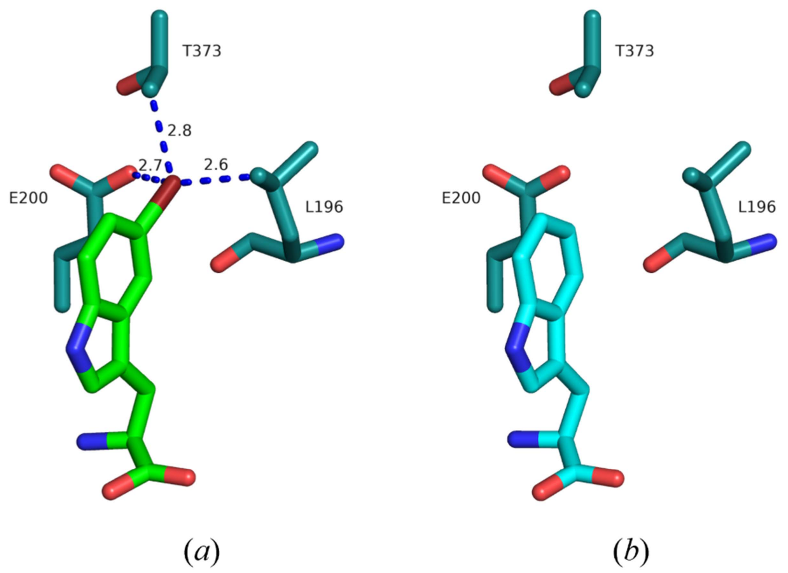

Figure S14 (*a*) 5-Br-Trp (green carbon atoms) superimposed on L-Trp in AetF–L-Trp. (*b*) L-Trp (cyan carbon atoms) in AetF–L-Trp (8cje) as reference. The blue dashed lines mark clashes of the C5 bromo substituent with surrounding protein atoms.

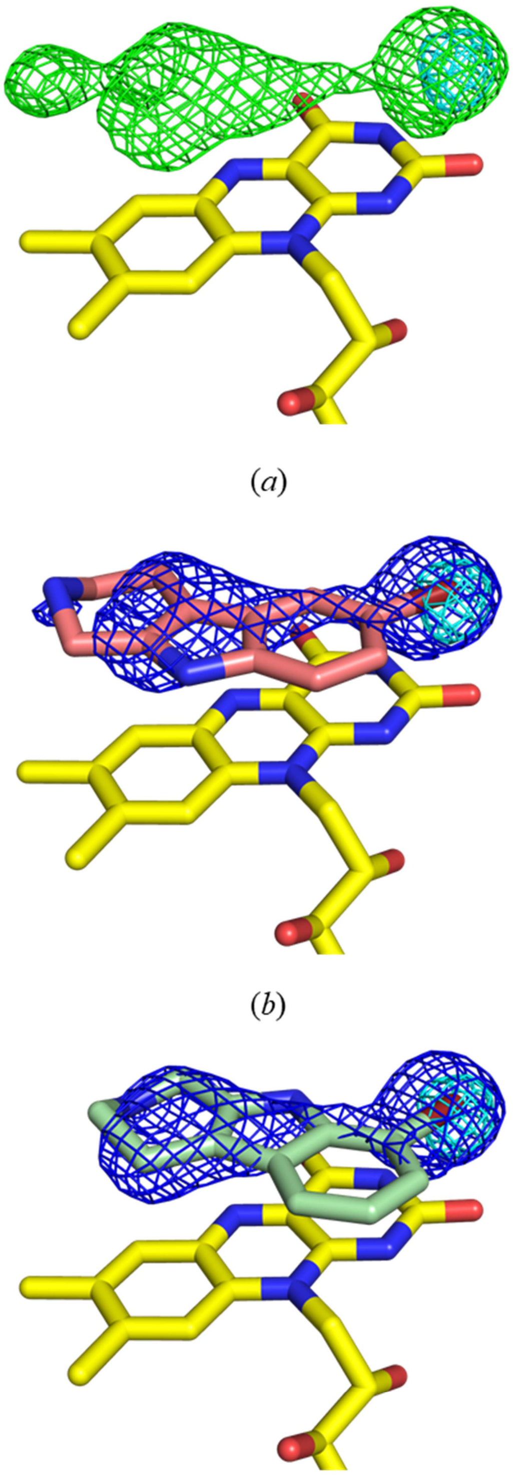

(*a*)

(*b*)

(*c*)

Figure S15 (*a*) Initial $mF_o - DF_c$ (green mesh, +3 σ) and anomalous density (yellow mesh, 5 σ) stacked on top of FAD (yellow carbon atoms) in Chain *A*. (*b*) $2mF_o - DF_c$ density (blue mesh, 1.5 σ) when modelled as 6-bromotryptoline (salmon colored carbon atoms). (*c*) $2mF_o - DF_c$ density (blue mesh, 1.5 σ) when modelled as 8-bromotryptoline (pale green carbon atoms).
